# Machine learning for the life-time risk prediction of Alzheimer’s disease: a systematic review

**DOI:** 10.1093/braincomms/fcab246

**Published:** 2021-10-21

**Authors:** Thomas W Rowe, Ioanna K Katzourou, Joshua O Stevenson-Hoare, Matthew R Bracher-Smith, Dobril K Ivanov, Valentina Escott-Price

**Affiliations:** 1 UK Dementia Research Institute, Cardiff University, Cardiff, UK; 2 Division of Psychological Medicine and Clinical Neurosciences, School of Medicine and Clinical Neurosciences, School of Medicine, Cardiff University, Cardiff CF24 4HQ, UK

**Keywords:** machine learning, AUC, SNPs, Alzheimer’s disease, EPV

## Abstract

Alzheimer’s disease is a neurodegenerative disorder and the most common form of dementia. Early diagnosis may assist interventions to delay onset and reduce the progression rate of the disease. We systematically reviewed the use of machine learning algorithms for predicting Alzheimer’s disease using single nucleotide polymorphisms and instances where these were combined with other types of data. We evaluated the ability of machine learning models to distinguish between controls and cases, while also assessing their implementation and potential biases. Articles published between December 2009 and June 2020 were collected using Scopus, PubMed and Google Scholar. These were systematically screened for inclusion leading to a final set of 12 publications. Eighty-five per cent of the included studies used the Alzheimer's Disease Neuroimaging Initiative dataset. In studies which reported area under the curve, discrimination varied (0.49–0.97). However, more than half of the included manuscripts used other forms of measurement, such as accuracy, sensitivity and specificity. Model calibration statistics were also found to be reported inconsistently across all studies. The most frequent limitation in the assessed studies was sample size, with the total number of participants often numbering less than a thousand, whilst the number of predictors usually ran into the many thousands. In addition, key steps in model implementation and validation were often not performed or unreported, making it difficult to assess the capability of machine learning models.

## Teaser

This review assessed the literature regarding the performance of machine learning for the prediction of Alzheimer’s disease using genetics. Publications were screened systematically, with 12 studies passing all criteria. Common issues such as the overreliance on one data source, insufficient sample sizes and inconsistent model performance reporting methods were outlined.

## Graphical Abstract

**Graphical Abstract:**
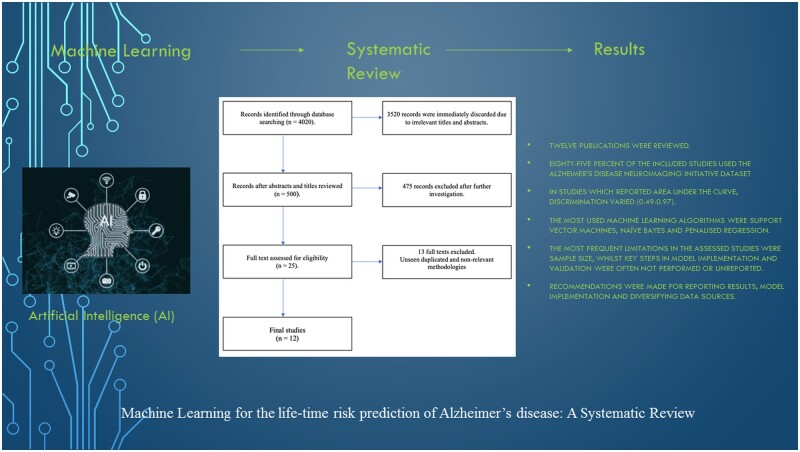

## Introduction

Dementia comprises a number of neurodegenerative disorders which cause a range of symptoms, some examples of these are memory loss, depression/anxiety and physical impairments such as incontinence.^1^ The most common form of dementia is Alzheimer’s disease, accounting for more than 75% of cases.^2^ The main neuropathological characteristics of Alzheimer’s disease are the accumulation of amyloid beta plaques and neurofibrillary tangles consisting of tau protein, which impact brain function.^3^

Diagnosing the correct form of dementia has long proven difficult due to different forms sharing phenotypic characteristics.^4^ Currently, the only method to confirm a diagnosis of a specific type of dementia, is post-mortem brain biopsy.^5^ Along with an individual’s age, genetics has been shown to be a strong risk factor for developing Alzheimer’s disease. Twin and family studies have suggested that up to 80% of Alzheimer’s disease involves the inheritance of genetic factors.^6^ However, Genome Wide Association Studies (GWAS) have failed to explain the level of heritability shown in twin studies.^7^ The GWAS-based heritability estimates assume an additive model, which, in statistical terms, is equivalent to looking for the main effects of common variants contributing to disease risk. In the genetics of complex diseases, it is unknown whether and to what extent non-additive genetic interaction effects contribute to risk.^8^ Risk prediction modelling is often used to assess an individual’s risk of developing a given disease.^9^ While there are currently no specific treatments to prevent Alzheimer’s disease or reverse its course, determining an individual’s risk of onset at an early stage can enable clinicians to improve quality of life during disease progression. This can be achieved through a combination of medication and palliative care, which are most effective when commenced in an early stage of the disease. Early prediction can also provide insights to patients and caregivers, enabling them to prepare for the personal implications of Alzheimer’s disease.^10^ This review assesses the use of genetic data to predict the risk of an individual developing Alzheimer’s disease at any time, or lifetime disease risk with machine learning (ML) approaches, which are suitable for detection of any effects contributing to disease risk, including non-linear effects.

ML can be defined as a set of algorithms which learn underlying trends and patterns in data. It is not a novel concept, however, interest in its applications has increased significantly in recent decades. This is due to modern computers being able to process larger datasets and perform in depth mathematical calculations in less time.^11^ Advantages of ML lie mostly in the ability of algorithms to learn from complex datasets, with emphasis on analysing hidden relationships which may be non-linear. Therefore, ML algorithms are able to provide data-driven classifications in a multidimensional space of predictors, instead of hypothesis-driven approaches testing a subset of predictors at a time.^12^

Advancements in biotechnology have resulted in various aspects of human biology being reliably recorded, including genetic data and other commonly used biomarkers, e.g. cerebral blood flow, brain imaging. This has led to the accumulation of large biological datasets which ML algorithms can learn from, with the aim of classifying the participants or predict the membership of predefined classes.^13^ The combination of genetic data with other data modalities often leads to complexity, which cannot be processed easily by humans in an un-biased way.^14^

However, despite the advantages of using ML for answering biological questions, possible issues must be overcome in the ML model development and implementation. Overfitting is a common issue when developing ML models,^15^ whereby a ML model does not generalize well from observed to unseen data. In this instance, while the model may perform well when making predictions on training data, predictions are not accurate when exposed to new data. Another relevant issue which may arise when using ML is insufficient sample size. The scenario in which the number of predictors is larger than the number of samples in a dataset often leads to optimistically biased ML performance.^16^ Genetic datasets are likely to fall into this category due to the many thousands of genetic markers in the human genome.^17^ Therefore, a careful and clear strategy for the validation of ML models must be considered in order to prevent overfitting and overinterpretation of the results.

This review assesses the ability of ML methods to predict lifetime risk for Alzheimer’s disease using primarily genetic [single nucleotide polymorphisms (SNPs)] data, however, studies in which SNPs had been combined with other forms of data were also considered. Initially, all forms of dementia were examined, however, searches returned publications focussed on Alzheimer’s disease only. The review was written in line with the Preferred Reporting Items for Systematic Reviews and Meta-analyses (PRISMA) guidelines.^18^ Databases were searched for relevant scientific articles, followed by an assessment on how prediction models were developed. Reviews in this area have been conducted previously^19^; however, this review is unique in its assessment for the possibility of bias for prediction models in this subject area, as well as in the number of ML methods that it includes. The risk of bias (ROB) was assessed by using the prediction model risk of bias assessment tool (PROBAST).^20^

## Materials and methods

### Search strategy

The online article databases Scopus, PubMed and Google Scholar were used to identify relevant publications for this review. Search terms used were ML, genetics, dementia, Alzheimer’s, SNP, polymorphism, mutation, variant and marker. These were used to retrieve studies published between December 2009 and June 2020. An initial search and screening for relevant publications was conducted by assessing both abstracts and titles. Based on eligibility criteria (listed below), publications from the initial search were then further assessed by two independent reviewers. Any discrepancies were then resolved by a third reviewer.

### Inclusion criteria

- Written in the English language
- Subject matter of Alzheimer’s disease
- The use of SNP data only, unless it was combined with other forms of non-genetic information.
- Supervised ML techniques
- Prediction resulting in a binary outcome (i.e. case/control)

### Exclusion criteria

- Prediction of Alzheimer’s disease related sub-phenotypes (e.g. MCI versus controls)
- The use of genetic variants other than SNPs as predictors. The search was deliberately broad (see Search Strategy section) to capture papers from non-genetic fields, which do not apply a refined definition of genetic variants

We identified articles published between December 2009 and June 2020. ML techniques have been used in studies prior to this time frame. However, interest in ML in biological research has increased mostly in the last decade^21^; therefore, studies previous to this were sparse and this recently defined window was used. SNPs were the only form of genetic variation accepted to facilitate comparisons between studies, therefore, articles focussing on gene expression data or other forms of genetic data (e.g. rare variants) were not included. Instances where authors had combined SNP data with other forms of predictive biological variables were included, e.g. MRI and PET. Only models which predicted a binary outcome between cases and controls were included, resulting in the exclusion of prediction models involving mild cognitive impairment (MCI). This was due to historic difficulties for clinicians to distinguish between MCI and Alzheimer’s disease status.^22^ Therefore, accepting models which discriminated between case and control status allowed a clearer assessment of the predictive performance.

For the purpose of assessing the suitability and comparability of ML approaches, prognostic and diagnostic models are usually considered separately. Prognostic models are defined as those which focus on future events and use longitudinal data, whereas diagnostic models are based upon current events using cross-sectional data. Limiting our search to binary outcomes only, revealed no prognostic models.

### Data extraction

The Critical Appraisal and Data Extraction for Systematic Reviews of Prediction Modelling Studies (CHARMS)^23^ was used as a tool to perform data extraction. CHARMS provides two tables of check points to be considered by the reviewer. The first table provides guidelines on how to frame the aim of a review, including how to search and filter extracted publications. The second table lists aspects to be extracted from each study for comparison, including predictor type, sample size and the amount of missing data. CHARMS also gives guidance on assessing how certain aspects were reported such as model development, model performance and model evaluation. Advantages of using CHARMS include replicability across different types of reviews, its ease of use and assisting reviewers in producing transparent publications.^23^

The ability of ML methods to discriminate between two classes was extracted independently from all studies by two authors. Accuracy (ACC) describes the performance of a classifier with respect to all samples, it is calculated as the number of correct predictions divided by the total number of predictions made. However, it does not provide information on how well the model performs within the positive and negative classes.^24^ Sensitivity is calculated by using observed positive outcomes to determine the proportion of classifications correctly made in the positive class, while specificity measures the same statistic in the negative class. Area under the receiver operating characteristic curve (AUC) represents the trade-off between these two measurements at different thresholds, aiming to find the optimal balance.^24^ AUC was extracted in order to draw comparisons between the studies. Confidence intervals for AUC were also extracted if provided, otherwise these were calculated using the Newcombe method.^25^ Precision can be defined as the ratio of correct predictions in the positive class, divided by the total number of positive predictions. Measures of performance such as accuracy, sensitivity, specificity and precision were also recorded alongside AUC if present. As the true positive rate and recall are different terms used for sensitivity, while specificity is also known as the true negative rate, they were categorized under sensitivity or specificity (if reported).

**Figure 1:**
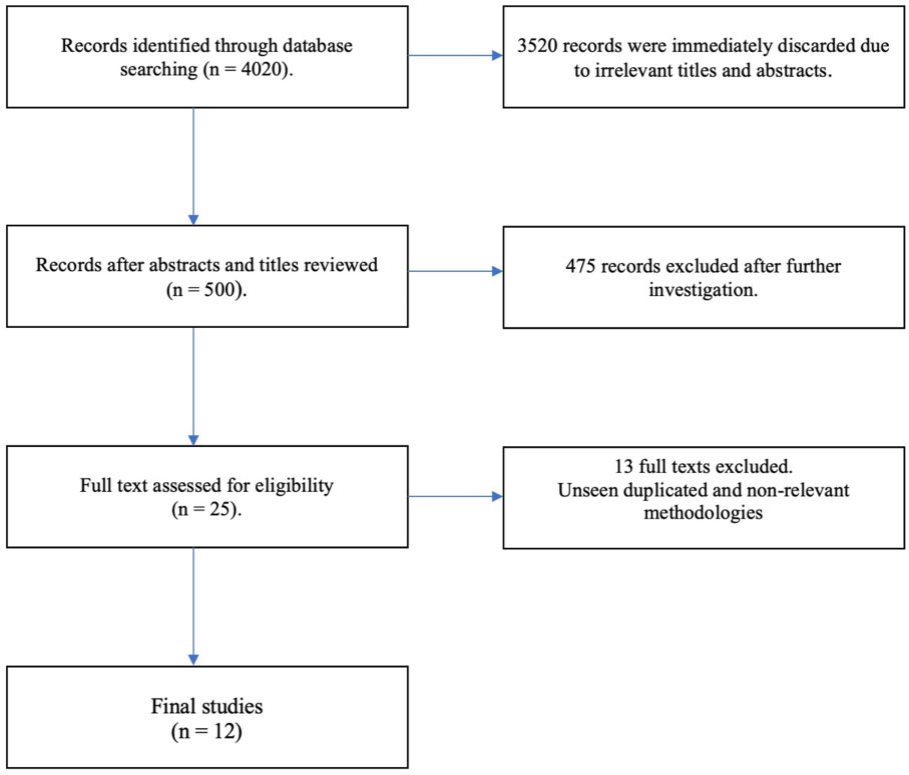
Visual breakdown of publication selection based on a similar diagram found in PRISMA.

**Figure 2:**
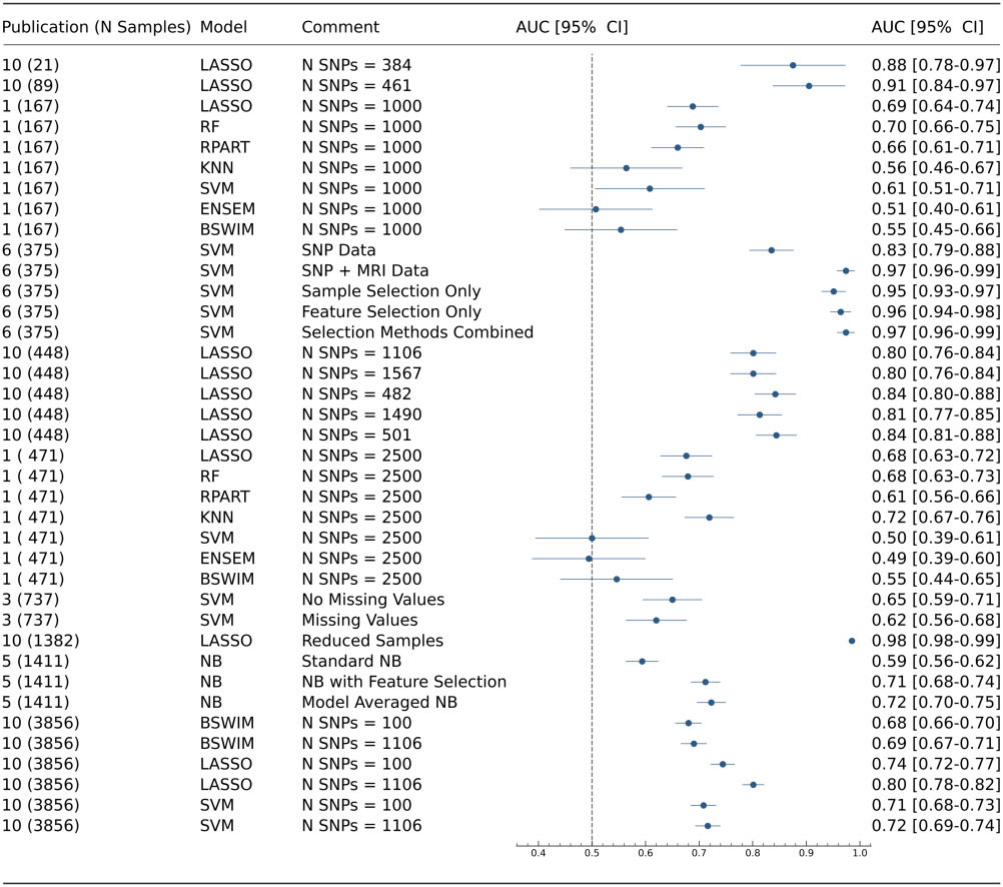
**A forest plot displaying models used across publications which reported AUC, with the addition of confidence intervals derive using the Newcombe Method.** Column 1—Publication number as found in Supplementary Table 1, along with sample size. Column 2—Type of machine learning model. Column 3—Information to help distinguish between models in publications, including differing SNP numbers and methodologies.

**Figure 3:**
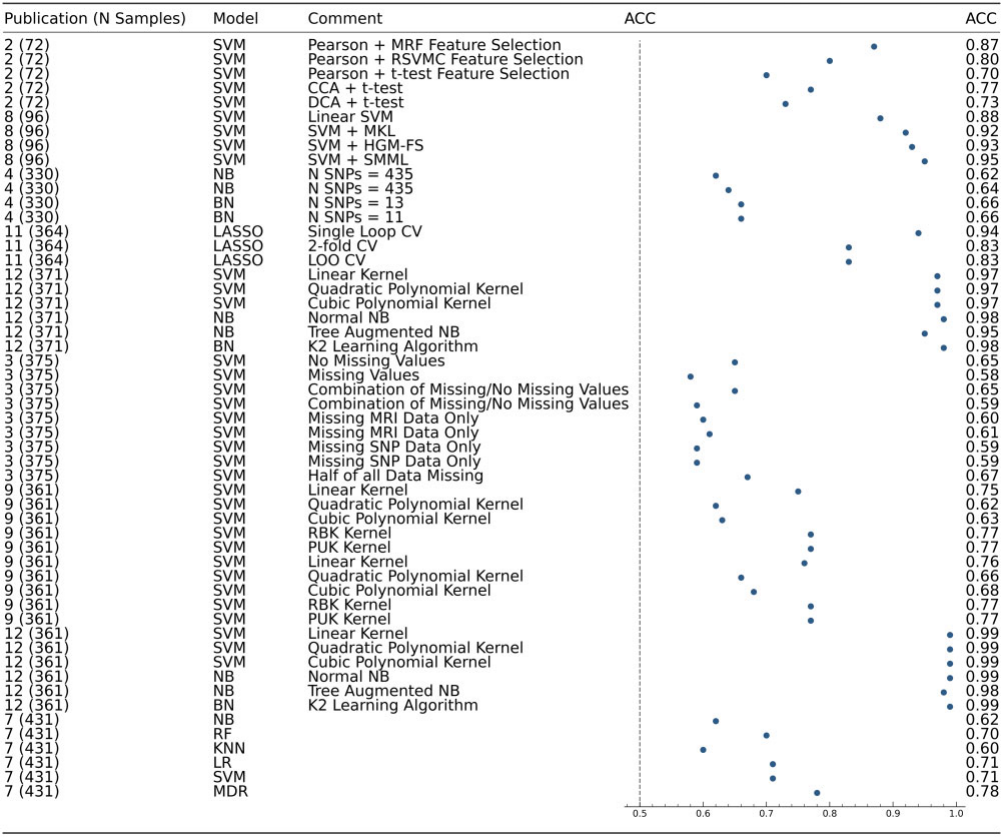
**A forest plot displaying all models used across publications which reported ACC.** Column 1—Publication number as found in Supplementary Table 1, along with sample size. Column 2—Type of machine learning model. Column 3—Information to help distinguish between models in publications, including differing SNP numbers and methodologies.

Statistics such as age and gender for participants, types of predictors and ML models were also extracted, as per the CHARMS checklist guidance. Figures in this study were created using Microsoft Word (Fig.  1) and the programming language Python (Figs  2 and 3).

Studies were analysed in order to determine whether they reported the calibration of their models. Calibration is defined as the accuracy of risk estimates and demonstrates how well predicted and observed probabilities of the class membership line up. Previous systematic reviews conducted for prediction models across a number of research areas have shown that calibration is rarely reported.^26^ Poor calibration could lead to healthcare professionals or patients having false expectations for certain events.^26^

### Data analysis

When assessing a number of studies in a review, meta-analyses are often conducted. A meta-analysis produces a weighted average of the reported measures, where the heterogeneity between studies is taken into consideration. If studies overlap, e.g. contain (partially) the same individuals, the resulting correlation between the studies will bias the results of the meta-analysis,^27^ unless taken into account. Since the majority of the extracted publications used the same dataset, a meta-analysis was not performed in this review.

ROB is another component to critically assess when conducting a systematic review of prediction models within studies. PROBAST uses a system of questions split over four categories: participants, predictors, outcome and analysis. Each category contains multiple choice questions assessing an occurrence of shortcomings in that category (with choice of answers from: ‘yes’, ‘probably yes’, ‘no’, ‘probably no’ and ‘no information’). If any question is answered with no or probably no, this flags the potential for the presence of bias, however, assessors must use their own judgement to determine whether a domain is at ROB or not. An answer of no does not automatically result in a high ROB rating. PROBAST does offer assistance on how to reach an overall conclusion on the level of bias in that category. In this review, we assessed all selected studies for ROB.

### Data availability

This review did not use or generate any form of new data.

## Results

### Search results

Following an initial search, a total of 4020 publications were returned. This number was reduced by assessing whether both titles and abstracts aligned with the inclusion criteria, resulting in 500 studies. A more in-depth analysis was then conducted on the full texts, removing publications which did not pass the inclusion criteria upon a detailed inspection, 25 texts remained at this stage. These were further reduced to 21 due to the presence of duplicates, comprising both pre-prints and conference abstracts. Nine further publications were then removed due to non-relevant methodologies, leaving a final set of 12 studies to be included. A visual representation of the selection process is given in Fig.  1.

The majority of publications (10/12) used the publicly available Alzheimer’s Disease Neuroimaging (ADNI)^28^ dataset. ADNI is a longitudinal study measuring various biomarkers in both Alzheimer’s disease cases and healthy age-matched controls. However, all studies reported here analysed a particular subset of the cohort at a fixed timepoint only. Therefore, only cross-sectional format data were used, and hence models throughout publications were classed as diagnostic rather than prognostic. Out of the publications using ADNI, four used the initial five-year study (ADNI-1), whilst the remaining studies did not specify which cohort was used. There were two studies that did not use ADNI. Wei et al.^29^ used a combination of three datasets^30^ in which biomarkers were collected at a fixed time point, therefore, data were cross-sectional. Romero-Rosales et al.^31^ used a longitudinal source of data known as the National Institute on Aging-Late-Onset Alzheimer’s Disease Family Study (NIA-LOAD).^32^ Again, values for predictors were taken at a fixed time point, thus the data used were cross-sectional. All models across the included studies were classified as diagnostic.

A range of ML approaches were used across the 12 reviewed studies. Table  1 outlines all types of models used and their frequency across the publications. The most commonly used ML approach across the analysed publications was Support Vector Machines (SVMs), followed by Naïve Bayes (NB) and Penalized regression. The number of tested models was also the highest for SVMs. This approach allows the most flexibility when adapting models via kernel functions.^33^ Penalized regression was commonly used in the form of the Least Absolute Shrinkage and Selection Operator (LASSO). This type of regularization shrinks coefficients closer to zero when compared to their maximum likelihood estimates and simultaneously reduces variance in predictions and performs predictor selection. These aspects make penalized regression a popular method in prediction analysis.^34^ Random forests (RFs) were also used across three studies, these algorithms are intuitive in their use of decision trees, are invariant to scaling, and provide an in-built measure of predictor importance, which likely explains their favour in biology.^35^Supplementary Table 1 outlines how the model types displayed in Table  1 were distributed across publications. It also provides study names, sample sizes and which methods were used to report results. The most commonly used statistics for model performance were ACC and AUC. With five studies reporting AUC and the remaining seven studies reporting ACC.

**Table 1:** Summary of ML methods used in the analysed publications

ML approach^a^	Number of publications^b^	Number of models reported across publications^c^	Additional information^d^
Support vector machine (SVMs)	8	44	Linear kernels (22 models, 5 studies). Quadratic polynomials (4 models, 2 study). Cubic Polynomials (4 models, 2 study). Radial basis functions (3 models, 2 studies). Pearson kernel function (2 models, 1 study). Unreported kernels (9 models, 3 studies). A supervised method which uses distance-based calculations to separate samples into groups.
Penalised regression (LASSO)	4	15	All 15 LASSO regressions across 3 studies. A regression analysis which performs both feature selection and regularization.
Naïve Bayes (NB)	4	10	Six ordinary NB models, three tree-augmented NB and one model averaged NB. A probabilistic classifier which uses bayes theorem to make predictions.
Random forest (RF)	3	5	Five classification RFs used, two of which used the RPART package. These are an ensemble of decision trees which produce aggregated classifications.
Bayesian networks (BN)	2	4	2 BNs with K2 learning algorithm, one markov blanket and one minimal augmented markov blanket. A graphical model which calculates conditional dependencies between variables using Bayesian statistics.
Linear models	2	4	Bootstrapping Stage-Wise Model Selection (BSWiMS). A supervised model-selection algorithm which uses a combination of linear models for prediction.
K nearest neighbour (KNN)	2	3	This is a distanced based algorithm which uses similarities in features to classify.
Ensemble methods	1	2	Ensembles are the use of a number of ML models, these arrive at a collective prediction result.
Logistic regression (LR)	1	1	A form of linear regression whereby the outcome is a categorical variable.
Multi-factor dimensionality reduction (MFDR)	1	1	A technique used to detect combinations of independent variables that influence a dependent variable.

a Type of machine learning model.

b The number of publications models were used in.

c The number of publications these models occurred in.

d Further information regarding the machine model used.

BN = Bayesian networks; RF = random forest; KNN= K nearest neighbour; LASSO= least absolute shrinkage and selection operator; LR= logistic regression; MFDR= multi-factor dimensionality reduction; ML= machine learning.

### Risk of bias

For diagnostic models, data sources with the lowest risk of ROB for participants are of the cross-sectional form. The publications which used the ADNI dataset assessed it in a cross-sectional format. This assertion is reinforced in Gross et al.,^36^ where ADNI is described as a cross-sectional study with longitudinal follow-up. A similar decision was reached when considering the two studies which did not use ADNI, Wei et al.^29^ and Romero-Rosales et al.^31^ After considering this, ROB was deemed low for participants.

The focus of PROBAST for predictors is to assist the researcher in determining whether the procedures for measuring biomarkers were equal for all members of the study. ADNI provides publicly available documents which outline the methods for biomarker collection. Predictors derived from blood samples or MRI scans were collected using the same protocols for all participants. Therefore, the process of collecting predictors was deemed to be of low ROB. Genotyping of SNPs for the NIA-LOAD dataset^32^ was performed in the same way across all samples, therefore, ROB for predictors was low for Romero-Rosales et al.^31^ Procedures for collecting predictors in Wei et al.^29^ were not provided. This was also the case when assessing the original source of the data by Romero-Rosales et al.^31^; therefore, ROB for predictors for these publications was stated as not known.

Blinding is the process whereby samples from patients are collected without prior knowledge of their disease status. Such knowledge has been shown to introduce bias to collection procedures.^37^ According to the ADNI data generation policy, samples were collected using blinding and only unblinded when uploaded to databases. Imaging data were collected and processed using standardized automated pipelines, thereby reducing the possibility of multiple clinicians using different methods when collecting predictors.^38^ ROB was deemed low for blinding in ADNI. Policies for blinding were not provided by either Wei et al.^29^ or Romero-Rosales et al.^31^ Therefore, a judgement could not be made for either publication.

ROB in the PROBAST category ‘outcome’ was considered to be low for the majority of studies. PROBAST’s questions regarding this section focus on how the outcome was determined and whether this determination was applied equally to all participants. ADNI used a range of clinically accepted methods to determine an individual’s Alzheimer’s disease status, including the Mini Mental State Examination and the Clinical Dementia Rating. The use of multiple methods of cognitive performance reduced the possibility of misdiagnosis, which in turn reduced the ROB. Diagnosing the outcome for participants in NIA-LOAD study was also achieved using a range of stringent methods. NINCD-S-ADRDA^39^ criteria were used for Alzheimer’s disease diagnosis at recruitment, while diagnosis was pathologically confirmed for participants who were deceased. Controls were determined using neuropsychological tests in which memory function was examined, coupled with examination for any previous history of neurological disorders. As methods for both controls and cases were applied uniformly across the study participants, with the exception of deceased and alive Alzheimer’s disease individuals, the ROB for Romero-Rosales et al.^31^ was deemed low for outcome. In Wei et al.^29^ all brain donors for cases satisfied clinical and neurobiological criteria for cases of late onset Alzheimer’s disease, while clinical cases satisfied criteria for probable Alzheimer’s disease.^40^ Also, brain donor controls did not have significant cognitive impairment at the time of death and clinical controls exhibited no cognitive impairment. However, the methods used to determine these diagnoses were not elaborated upon. For instance, whilst there was a mention of using clinical criteria, these were not defined. Therefore, ROB for outcome was unclear.

The fourth and final category in which PROBAST aids investigation is in the analysis phase of a study. All studies exhibited high ROB for this section, with a consistent lack of reporting for calibration; additionally, 5 out of 12 publications did not report possible missing values in their data and how these were dealt with if present. To assess whether sample sizes used in modelling are adequate, PROBAST suggests the use of the metric Events per Variable (EPV). EPV is defined as the number of events in the minority class (i.e. the smaller of either cases or controls), divided by the number of candidate predictors used. In cases where more in-depth algorithms [e.g. Neural Networks (NNs)] are used, model parameters are also included in the calculation of EPV. We evaluated ROB using a value of at least 10 EPVs, following common recommendations.^16^ However, this threshold may be tailored more to the accurate estimation of regression coefficients in a logistic regression model. More complex algorithms which require the tuning of hyperparameters (RFs, SVMs, NNs) may require a value of over 100.^41^ Values across all studies were assessed to be below this threshold. The study with the highest EPV of 9.43 was Chang et al.^42^ The lowest EPV, 0.0018, was found for Wei et al.^29^

Values of EPV below the recommended threshold of 10 introduce the possibility of overfitting, which in turn could result in spurious results.^16^ However, efforts were made by most studies to overcome the problem of overfitting, mostly in the form of cross-validation (CV) (11/12 studies). During this process, the data are divided into *k* partitions, with *k*-1 partitions used as training data and the remaining partition used as the test set. This process is then repeated *k* times. It has been demonstrated that using CV is a viable method for authors to address overfitting.^43^ Despite this, the possibility of bias could still be present if the correct form of CV is not used. To investigate the importance of CV type selection, several methods of CV were used on datasets with low EPV values.^44^ The simplest form of CV *(k*-partitioning) was shown not to counteract the issue of overfitting in some instances and could even exacerbate the problem. Nested-CV has been shown to achieve the best performance of all methods^45^ and it operates by using an outer and inner loop of CV. The outer loop splits *k* times to perform model validation while hyperparameters and feature selection are conducted in the inner loop. This method was only reported by two of the included studies.^46^

### ML performance

Figures  2 and 3 summarize the reported accuracies across all included studies and ML methods. The first column shows the reference number of the publication as listed in Supplementary Table 1, along with the sample size used in the respective ML model. ML approaches used are shown in the second column. The third column displays information which assists the reader in distinguishing between models in the same study, this includes factors such as number of SNPs used, and methodologies implemented. Studies were sorted by sample size in ascending order. The vertical dashed line shows the accuracy of 0.5, which indicates a 50% chance of the result being correct. The last column shows the actual values of the accuracy achieved. Confidence intervals of AUC values in Fig.  2 were calculated using the Newcombe method.^25^ These confidence intervals reflect the variability of AUC controlling for sample size. This allows for comparison between studies with large sample size differences. If the intervals overlap between studies, then the AUCs are not significantly different between models.

Five studies recorded AUC for the performance of models, ranging from 0.49 to 0.97. The remaining seven studies reported mainly ACC, sensitivity and specificity (Supplementary Table 2). The highest AUC value was achieved by An et al.^47^ (Study 6 in Fig.  1), where the authors used a hierarchal method to find the optimal set of features for the prediction of Alzheimer’s disease. Manifold regularization was used to combine both genetic and MRI data in a semi-supervised hierarchal feature and sample selection framework. This method utilized both labelled and unlabelled data in order to maximize the amount of information for prediction. For classification purposes, SVMs were used to discriminate between controls and cases. However, the EPV score was 0.919 and this is below the recommended threshold of 10. This could introduce the possibility of overfitting which can in turn lead to spurious results.^16^ The authors used CV to alleviate the potential for overfitting.

A single study reported calibration statistics^29^ (Publication 5 in Supplementary Table 1). The authors compared the predictive capability of a model using averaged NB with both standard NB and NB with feature selection. The method used to report calibration was calibration curves. The results highlighted that the model using averaged NB achieved better calibration than the standard NB model and achieved similar performance to the NB with feature selection. The prediction accuracy of these models was 0.59–0.72 (Publication 5 in Fig.  1).

Ten-fold CV was the most common form of validation used, however, a range of other values of *k* were also documented. One further study used a nested CV approach to optimize both model performance and hyperparameter tuning. Leave one out CV was also used by one study, this functions by creating a number of folds equal to the number of data points in the training set. Within each fold a single data point is removed to be used as the test set, the algorithm is then trained on the remaining points. Prediction performance is calculated by averaging over the results for all folds. Also, one publication explored a different approach of dividing the data into training and test datasets called a split sample. In this process, a model is trained using a training set and is subsequently tested on a validation (test) set, where the test dataset contains the remainder of the original data not included in the training dataset. All of these methods are known as internal validation, where model optimization and hyperparameter tuning is achieved using a single dataset. External validation involves using a completely separate cohort to validate an already trained model, usually this cohort has been independently gathered and assessed to the initial training data.^48^ This method was not used by any study in this review (Supplementary Table 3).

### Sample size

**Figure 4:**
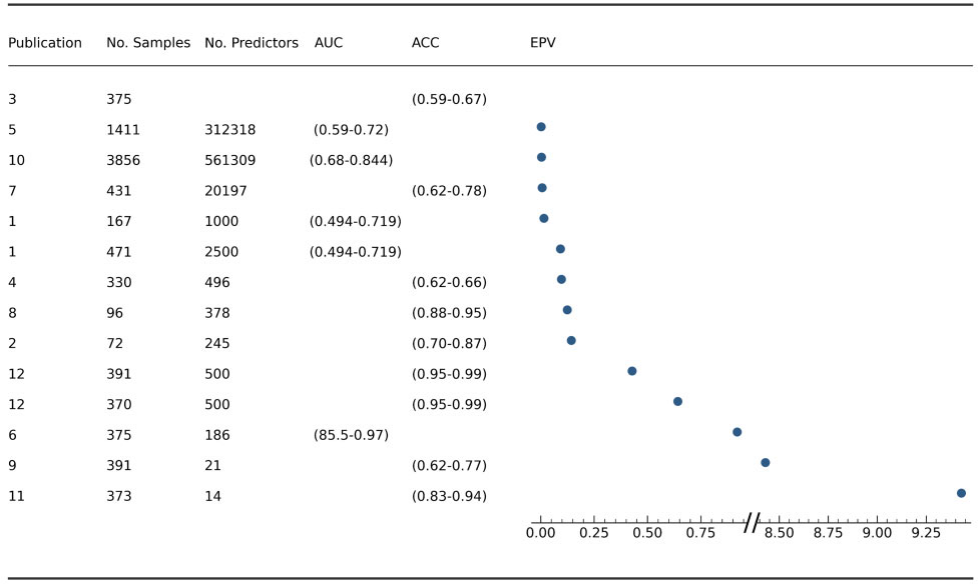
**A forest plot displaying all available EPV values across the included studies.** Column 1—Publication number as found in Supplementary Table 1. Column 2—Number of samples. Column 3—Number of predictors used, Column 4—AUC of models if reported, Column 5—ACC of models if reported, Column 6—values of EPV.

Sample sizes ranged from 72 to 3856 individuals, with the largest cohort being the NIA-LOAD dataset.^49^ The majority (10/12) of studies used 300–900 individuals from the ADNI dataset. The number of SNPs used in models varied between studies, with numbers ranging from 21 to 561 309 SNPs. The large range in the number of SNPs used was due to differences in the used methodologies. The study which used the greatest number of SNPs^31^ investigated improving AUC by reintroducing initially misclassified samples to the final models. The study which used the least number of SNPs focussed only on the top 10 genes associated with Alzheimer’s disease,^50^ thereby limiting the number of SNPs included in the study. EPV ranged from 0.0018 to 9.43 for eleven studies, with one study not providing enough information to calculate EPV. These values are displayed in Fig.  4, this also includes the number of samples, amount of predictors used and values of either ACC or AUC for each study. The publication number corresponds to those used in Figs  2 and 3. Owing to the large difference between two values and the rest, two scales were used to allow for all points to be plotted on the same figure. Imbalances between classes, as a ratio between controls over cases, ranged from 0.408 to 6.55, with a median value of 1.193 (Supplementary Table 4). The accuracy for the study with the highest class imbalance (6.55) was 0.95–0.99 ACC.^51^

### Predictors

Criteria used for inclusion specified that SNPs were the only form of genetic data used as predictors. However, other predictors were also considered, whereby other forms of predictive material were used alongside SNPs. The most common form of secondary data used was MRI, included in four publications. PET imaging data were also used in two studies. Additionally, CSF was used in one publication (Supplementary Table 5).

Pre-processing techniques for SNPs were reported in the majority (10/12) of studies. All these studies excluded SNPs which did not satisfy Hardy-Weinberg equilibrium.^52^ SNPs were selected with a variety of Alzheimer’s disease association significance thresholds (0.00007–0.05), leading to different numbers of SNPs being retained across studies. Seven of the studies which discussed pre-processing for SNPs also used minimal minor allele frequency (MAF), i.e. rare variants were removed from a SNP set based on their allele frequency. Thresholds used for MAF varied (0.01–0.04) across studies (Supplementary Table 5). Two studies did not report steps taken to pre-process SNPs; this could lead to questions regarding data quality.

Eight out of 12 studies used methods to address missing data values. Two studies excluded samples with >10% missing predictor values. A further four publications described processes for the imputation of missing genotypes. For instance, Sherif et al.^53^ imputed missing SNP values by using the expectation maximization algorithm. Another study^31^ imputed missing genotypes by using the median value of the nearest neighbours, this was the only example of using a measure of central tendency. Zhou et al.^46^ did not remove or impute missing data, rather they designed a method in which samples with missing values were incorporated in the models. All complete samples were used to develop a latent representation space. Samples with missing values were used to learn independent modality specific latent specifications. These latent representations were then used as an input for the Alzheimer’s disease classifier. This process allowed these authors to produce models which outperformed comparable methods of dealing with missing data and selecting features.

None of the analysed studies which reported the use of imputation methods specified whether this process was undertaken before CV or afterwards, which may be prone to the issue of data leakage.^54^

### Hyperparameter search

Hyperparameter tuning is a common step in developing prediction models, it is implemented to ensure the optimization of AUC.^55^ Reporting of techniques for hyperparameter optimization was inconsistent across studies, with seven publications not providing values or the process of tuning. For the remaining five studies, a range of differing techniques were used. Zhou et al.^46^ used a nested approach to optimize model parameters. Ten-fold CV was used to fit models, whilst an inner loop of 5-fold CV trained model hyperparameters. However, this was only the case for some hyperparameters, as some were fixed at pre-determined values to reduce training times. This arbitrary fixing of values could introduce bias. Hao et al.^56^ also used a nested approach for hyperparameter tuning. Five-fold CV was used to optimize parameters for regularization, with a separate loop of five-fold CV used for model validation. These were the only two studies which reported the use of nested CV for hyperparameter tuning. The remaining 3 studies reported hyperparameter optimization but did not specify whether a nested approach was used.

Bi et al.^57^ used an iterative process to determine the optimum number of decision trees to use in their RF approach. Furthermore, grid search and CV techniques were employed to optimize varying hyperparameters across the studies (Supplementary Table 6, last column). In this process, CV is used to test different combinations of hyperparameter values, with the aim of producing the set which leads to the highest value of AUC. Seven publications did not report optimization methods. Of these seven studies, four used NB methods, which do not require hyperparameter tuning. For the remaining three studies, hyperparameter tuning was required but not reported.

### Descriptive statistics

Eight studies did not report values regarding both age and gender for study participants. The remaining four reported the age and gender distributions in both classes (cases and controls). De Velasco Oriol et al.^58^ reported age and gender for both the discovery and validation sets. Values for the mean age for both cases (75.4–75.5) and controls (76.1–77.4) were similar across studies. This similarity is due to the consistent use of the ADNI dataset throughout the analysed studies. The proportion of males to females in controls ranged from 0.59 to 1.22; in cases this proportion ranged from 1.05 to 1.22 (Supplementary Table 7).

## Discussion

This review assessed a selection of studies which used ML to predict Alzheimer’s disease from mainly genetic data. Using a systematic approach (PRISMA), 12 studies were identified which met inclusion criteria. This could be perceived as a low number of studies; however, this amount is consistent with other ML reviews.^59^ A potential reason for this small number is that ML is a relatively novel technique in Alzheimer’s prediction. Also, the disease risk associated with SNP data in complex genetic disorders has gained recent interest due to the appearance of GWAS, followed by prediction using polygenic risk scores.^60^ In addition, difficulties exist in accessing datasets with sufficient sample size for prediction. These manuscripts were reviewed to identify the type of models used, model development and the validity of the reported results.

AUC results in the included studies (5 out of 12) varied (0.49–0.97) for Alzheimer’s disease risk prediction. The most accurate models were shared across two studies, with the authors recording AUC >0.8, which could be considered as high (e.g. approved clinical prediction models in cardiovascular disease and diabetes typically achieve AUCs of 0.8–0.85^61^). Given that genetic prediction for complex traits is bounded by heritability and the disease prevalence,^62^ these results match and outperform the theoretical maximum prediction accuracy in AD using Polygenic Risk Scores (AUC = 0.82, assuming SNP-based heritability *h*^2^ = 0.24 and life-time disease prevalence of 2%^63^). Seven out of 12 publications did not report AUC for their models, with accuracy and sensitivity being the preferred choices. The most common measure of performance used other than AUC was ACC. Four studies reported ACC >0.8, which is considered important when attempting to reduce the possibility of miss-communicating risk to clinicians and the public. However, ACC can be skewed by the presence of class imbalances.^64^ In addition, ACC is calculated from all predictions against all observed outcomes, although this does not clarify how the model performs per class. For these reasons, we advocate that AUC should be used as a standard measure for reporting performance.

Continued research and development in the field of ML has led to an increasing number of algorithms available for use in risk prediction.^65^ This is reflected in the use of 10 different types of approaches across all studies, the most popular of these being SVMs. SVMs are known for their simple application and predictive accuracy, and are therefore used regularly in prediction modelling.^66^ Other notable algorithms used in the assessed studies were RFs and NB. Similar to SVMs, NB is known for its easy implementation. However, its performance can be hindered due to correlations between features used for prediction, which negates the naïve assumption that all input features are independent.^67^ If correlation between features is present, the importance of these features will be overemphasized during modelling.^68^ RFs, used in three studies, are a popular classifier due to their ability to negate overfitting. However, applying RFs to prediction problems can be challenging due to the need for hyperparameter tuning.^69^ Given the success of the forementioned algorithms in a range of application areas, it is perhaps not surprising that these three algorithms were the most used across all publications.^70^

None of the included studies used NNs to predict Alzheimer’s disease. NNs are powerful predictive algorithms, with the ability to learn non-linear patterns in complex datasets. In some scenarios, they can infer relationships in the data which are beyond the scope of other ML techniques.^71^ A possible explanation for their absence could be the structure of datasets used across the selected models, where the number of predictors often outnumbered individuals. In the scenario where a dataset has many more predictors than individuals, a prediction algorithm is more susceptible to overfitting.^72^ NNs are known for being complex to implement, as well as difficult for hyperparameter tuning and susceptible to overfitting.^73^ This could explain why they were not present in the reviewed studies.

Another potential reason for the absence of NNs in this review is the omission of the term from our keyword search, that is we searched for the term Machine Learning, rather than specific ML techniques. This could be purported as the main limitation of this review as some research papers might have been mistakenly excluded. A subsequent search for the use of NNs for Alzheimer’s disease prediction returned a study,^74^ which used deep NNs to predict Alzheimer’s disease from SNP data. Using the ADNI dataset, the authors conducted several experiments to predict case–control status. A standard architecture was implemented for the NN, along with 5-fold CV for model validation. Results for the NN across experiments centred around 65% AUC. However, this paper would not have been included in the review due to it being a pre-print, and therefore lacking a peer review.

A secondary study using NNs was also found, that used SNPs and MRI data from ADNI.^75^ The authors developed a novel stage-wise deep learning framework, which fused multimodal data in stages. This method achieved a classification accuracy of 64.4%.

Greater focus in recent years has been given to the possibility of bias when authors introduce novel concepts. For instance, authors may aim to achieve the best prediction accuracy possible in order to supersede previous publications. This may have been achieved by choosing datasets which produce the best accuracy only, leading to a lack of generalization in the research area. This possibility has led to comparative studies which draw comparisons between novel techniques and historic models.^76^

A number of consistent issues were highlighted across the included studies. One of the main focus points was the widespread usage of the ADNI dataset, where 10 of the 12 included studies used this as a data source. Methods used to demonstrate model performance were reported inconsistently. The combination of low EPV values and inconsistent model performance reporting led to the possibility of bias in the analysis phase of modelling.

In terms of model implementation, the main aspects scrutinized were the use of any hyperparameter tuning, as well as the methods used for model validation. Hyperparameter tuning has become an increasingly important part of ML development. The majority of algorithms require certain values for hyperparameters which are specified by the user. If these values are not optimized, then the model is susceptible to overfitting and inaccurate predictions.^77^ Five out of the 12 studies referenced the use of hyperparameters, the remaining 7 studies did not outline any tuning methods. Greater transparency about the use of hyperparameters and their tuning allows the reader to understand whether issues such as overfitting were accounted for. Therefore, researchers should report both hyperparameter values and methods used to obtain them.

Model validation is also an important aspect of predictive analysis. Correct methods of validation reduce the likelihood of overfitting, whereby algorithms become too reliant on the training/test data and cannot perform sufficiently when tested on unseen data.^44^ The most commonly used method among the selected studies (11/12) was CV. This method has become increasingly popular in prediction models, due to its ability to counteract overfitting.^78^ Eleven of the 12 studies which reported CV used a varying number of folds, whilst one of these publications used a technique called leave one out CV. In the majority of cases, the higher the number of folds, the greater the accuracy from CV. However, increasing the number of folds leads to a higher chance of overfitting.^78^ Therefore, leave one out CV is only suitable for small datasets, where the number of samples is <100.^79^ Nested CV was used by two studies only. These were the only evidence of using separate validation folds for both model optimization and hyperparameter tuning throughout all included studies. Using the same CV split for both of these tasks can introduce overfitting,^45^ therefore we recommend the use of nested CV for future analysis. The only publication which did not report CV used a train and test split method for internal validation. The model is trained only once, increasing the chance of a model becoming too reliant on the training data and thereby reducing its ability to replicate in independent datasets. Since the split of the data is conducted randomly, an argument could be made that the derived results could be influenced by this single split.^80^ Therefore, methods which use a form of CV are recommended.

Calibration compares the similarity of probabilistic predictions with observed outcomes. This metric was only reported in one study.^29^ Calibration is of high importance when assessing ML performance, this is especially true when considering models which may be implemented in the medical sector.^81^ The implications of incorrectly communicating the risk of developing Alzheimer’s disease to an individual could cause considerable harm, by means of both physical and psychological trauma. With the potential of causing death due to incorrect treatment in the most serious of circumstances.^82^ Therefore, we recommend that authors aim to produce highly calibrated models and also report calibration statistics.

Another aspect investigated in this review was the sample size used in the training of models. These were relatively small with most studies using between 300 and 900 individuals (due to the common use of the ADNI dataset). Different quality control techniques also resulted in the number of predictors (SNPs) to vary across publications, ranging between tens of SNPs to over 100 000. The combination of small number of samples and large number of predictors led to low EPV scores, the highest of which was 9.43 in Chang et al.^42^ The common use of ADNI also contributed to low EPV values due to the consistent implementation of small numbers of participants and high numbers of predictors. A more commonly known term for low EPV values is the ‘curse of dimensionality’. This refers to the requirement for more training data when the number of features is increased. If the number of samples is not sufficient with respect to the number of features present, an ML algorithm is more likely to overfit. The number of samples, therefore, must increase at a certain rate in order to balance this relationship. Low EPV values suggest this balance has not been achieved.^83^

One method for dealing with a large number of features and the issues that this could cause, is feature selection. An example of this is Minimum Redundancy Relevance (mRMR). This method is widely used in genetic studies.^84^ In mRMR, features which are significantly correlated with the target variable are identified and this subset is then filtered further based upon correlations between features, with heavily correlated features being discarded. However, this method was used in only one^58^ of the 12 studies reviewed. To summarize, all EPV scores were below the threshold recommended by PROBAST. Small sample size may be a difficult issue to overcome therefore, it is advisable to use CV to reduce the impact of possible overfitting. Further techniques, such as nested CV have been shown to mitigate overfitting more effectively.^44^ We therefore encourage authors to investigate which type of validation technique would be suitable for their models.

This review aimed to assess ML models which used SNP data for Alzheimer’s disease prediction. Of the 12 studies reviewed, eight used SNPs only, and the remaining four combined SNPs with other data modalities. In terms of AUC, it appears that using a multimodal approach may lead to better prediction performance. The details are presented in Supplementary Table 1. For example, An et al.^47^ have shown that AUC was 85.5% for SNPs alone and 97.4% when both SNP and MRI data were considered together. However, for the studies that reported ACC only, there appears to be little difference in predictive performance between those which used SNPs only and those which used a multimodal approach.

When considering other factors which may cause differences in prediction performance, class imbalances appeared to have a negligible effect. Extreme values of class imbalance did not lead to largely different accuracy results. Class imbalances can lead to poorer prediction due to the model favouring the majority class. Techniques such as under/over sampling can be used in order to overcome this issue. Between the two methods, under sampling has been found to be more effective in addressing predictive bias.^85^ This is due to a common issue amongst over sampling algorithms, in which the creation of synthetic minority samples can introduce noise to the data.^86^ The issue of class imbalance was not of major concern in the reviewed papers, however with the availability of large population cohorts (e.g. UK Biobank), care should be taken when analysing diseases with small prevalence, which includes Alzheimer’s disease and other dementias.

Data leakage is another issue to be considered. It occurs when an algorithm’s performance is artificially inflated due to information being leaked from the training to test dataset. Manipulating data before training and validation may inadvertently leak information and boost performance. A way in which this can occur is pre-processing on the entire dataset before data is split. This is relevant to imputation of missing values, derivation of and adjustment for population structure. In order to avoid this, any pre-processing steps should be carried out separately in both the training and test datasets.^54^ To achieve non-biased results, an ML algorithm should always be validated on data separate to training data. Nested CV can be used to ensure pre-processing is carried out per fold, as this reduces the risk of data leakage.^87^

ROB in the remaining three sections of PROBAST (participants, predictors and outcome) was considered to be low for all publications. The usage of cross-sectional data reduced the ROB for the study participants. The use of a well-documented dataset (ADNI) provided details in areas such as predictor collection, the determination of disease status and inclusion of individuals in these studies. These areas could not be assessed in the two studies which did not use ADNI. The widespread use of ADNI also provided the possibility of comparison between studies due to the common data samples, however this prevented the possibility of performing a meta-analysis. The use of a range of data sources in future studies would be beneficial for the development of ML models and is likely to improve their robustness and replicability. In particular, the continued use of the same resource does not provide insight into the performance of ML in different populations. If used in frontline medicine, models will have to be able to predict upon individuals from different genetic backgrounds.^88^ For instance, 93% of the participants of ADNI are Caucasian.^28^ It has been shown that GWAS results from primarily Caucasian subjects do not replicate well in other races, which may also impact the prediction success of ML algorithms trained on them.^89^ Overall, despite ROB being low for the first three sections of PROBAST, issues within the analysis phase of modelling introduced possibilities of bias. This could bring the validity of the results into question.

Reviews in the field of ML for AD prediction have been previously conducted. Tanveer et al.^90^ conducted a comparison between three different ML techniques (SVMs, NNs and ensemble methods). The type of data used was imaging only, leading to a greater number of included texts. Comparisons were drawn between the methods but further detail on ROB was not included. Khan and Usman^91^ also conducted a review into ML prediction for dementia which included models using imaging data. In their review a large percentage of the studies used ADNI as their data source, and their results and conclusions follow a similar pattern to this review, however the authors did not formally assess ROB.

This review has highlighted a number of areas which require improvement in the field of ML for Alzheimer’s disease prediction using genetic data. Some areas require greater attention than others, namely the reporting of model performance and development. Reporting these measures thoroughly will allow for an accurate comparison between studies and provide better clarity for the performance of the models. More detailed description is also required when explaining model implementation, with special emphasis on hyperparameter tuning. This will provide greater understanding of how authors have attempted to maximize performance and reduce the possibility of overfitting. Furthermore, the majority of studies in this review used the publicly available ADNI dataset, which demonstrated a clear overreliance on one particular data source of Caucasian origin. Using a wider range of data sources would enhance the validity of results and also develop understanding of the applications of ML for Alzheimer’s disease prediction in more diverse populations.

In conclusion, ML will continue to be used more extensively in both academia and the industry due to its ability to analyse complex patterns in datasets, which will allow users to achieve better risk prediction as compared to more classical statistical methods. The continued usage of ML will boost the development of feature selection techniques and lead to improvements for classification and model optimization algorithms. These models have great potential to improve clinical risk prediction for Alzheimer’s disease, and many other complex genetic diseases. Since genetic data are classed as sensitive data under General Data Protection Regulation, most of the large genetic datasets require strict permissions and exact description of usage. UK Biobank is one of the largest cohorts, however it may not be suitable for application of ML to AD, as it is a population-based cohort with relatively young participants. The Dementias Platform UK (DPUK)^94^ is an attempt to provide a secure computational platform collecting genomic data from UK cohorts suitable for dementia research. The future of artificial intelligence applied to large genomic data lies with specifically designed secure computing facilities to store and analyse the sensitive data.

## Supplementary material

Supplementary material is available at *Brain Communications* online.

## Supplementary Material

fcab246_Supplementary_Data

## Abbreviations

ACC =: accuracy
AUC =: area under the receiver operating characteristic curve
ADNI =: Alzheimer’s Disease Neuroimaging
BN =: Bayesian network
CV =: cross-validation
EPV =: events per variable
KNN =: K nearest neighbour
LASSO =: least absolute shrinkage and selection operator
ML =: machine learning
MAF =: minor allele frequency
NB =: Naïve Bayes
NIA-LOAD =: National Institute on Aging-Late-Onset Alzheimer’s Disease Family Study
NNs =: neural networks
PROBAST =: prediction model risk of bias assessment tool
PRISMA =: Preferred Reporting Items for Systematic Reviews and Meta-analyses
RF =: random forest
ROB =: risk of bias
SNP =: single nucleotide polymorphism
SVM =: support vector machines
CHARMS =: The Critical Appraisal and Data Extraction for Systematic Reviews of Prediction Modelling
