# Supplementary material for: Machine learning for the life-time risk prediction of Alzheimer’s disease: a systematic review
Source: Brain Commun. 2021 Oct 21;3(4):fcab246. doi: 10.1093/braincomms/fcab246 (PMC8598986; doi:10.1093/braincomms/fcab246)
Supplement: fcab246_Supplementary_Data [file fcab246_supplementary_data.zip › Original Manuscript.pdf]

OXFORD UNIVERSITY PRESS | Brain Communications

Machine Learning for the Risk Prediction of Alzheimer's Disease: A Systematic Review

Journal:	*Brain Communications*
Manuscript ID	BRAINCOM-2021-086
Manuscript Type:	Review Article
Date Submitted by the Author:	11-Mar-2021
Complete List of Authors:	Rowe, Thomas; Cardiff University, Medicine<br>Katzourou, Ioanna; Cardiff University, Medicine<br>Stevenson-Hoare, Joshua; Cardiff University, Medicine<br>Bracher-Smith, Matthew; Cardiff University, Medicine<br>Escott-Price, Valentina; Cardiff University, MRC Centre for Neuropsychiatric Genetics and Genomics<br>Ivanov, Dobril; European Bioinformatics Institute; Cardiff University, Dementia Research Institute
Keywords:	Machine Learning, AUC, SNPs, EPV, Alzhiemer's disease

SCHOLARONE™ Manuscripts

Machine Learning for the Risk Prediction of Alzheimer's Disease: A Systematic Review.

Thomas W. Rowe*, Ioanna K. Katzourou*, Joshua O. Stevenson-Hoare, Matthew R. Bracher-Smith, Dobril K. Ivanov+, Valentina Escott-Price+

* - joint first authors

+ - corresponding authors

For Review Only

Abstract

Alzheimer's disease is a neurodegenerative disorder and the most common form of dementia. Early diagnosis may assist interventions to delay onset and reduce the progression rate of disease. We systematically reviewed the use of machine learning algorithms for predicting Alzheimer's disease using genetic data. Reviewers evaluated the ability of machine learning models to distinguish between controls and cases, while also assessing their implementation and potential biases. Articles published between December 2009 – June 2020 were collected using Scopus, PubMed and Google Scholar. These were systematically screened for inclusion leading to a final set of 12 publications. Eighty-five percent of the included studies used the Alzheimer's Disease Neuroimaging Initiative dataset. In studies which reported area under the curve, discrimination varied (0.49-0.97). However, more than half of the included manuscripts used other forms of measurement such as accuracy, sensitivity and specificity. Model calibration statistics were also found to be reported inconsistently across all studies. The most frequent limitation in the assessed studies was sample size, with the total number of participants often numbering less than a thousand, whilst the number of predictors usually ran into the many thousands. In addition, key steps in model implementation and validation were often not performed or unreported, making it difficult to assess the capability of machine learning models.

Keywords:
Machine learning, AUC, SNPs, Alzheimer's disease, EPV

Abbreviations:

Accuracy (ACC)

Area Under the Receiver Operating Characteristic Curve (AUC)

Alzheimer's Disease Neuroimaging (ADNI)

Bayesian Network (BN)

Cerebrospinal Fluid (CSF)

Cross-Validation (CV)

Events per Variable (EPV)

K Nearest Neighbour (KNN)

Least Absolute Shrinkage and Selection Operator (LASSO)

Logistic Regression (LR)

Machine Learning (ML)

Magnetic Resonance Imaging (MRI)

Minor Allele Frequency (MAF)

Multi-Factor Dimensionality Reduction (MFDR)

Naïve Bayes (NB)

National Institute on Aging-Late-Onset Alzheimer's Disease Family Study (NIA-LOAD)

Neural Networks (NNs)

Prediction Model Risk of Bias Assessment Tool (PROBAST)

Preferred Reporting Items for Systematic Reviews and Meta-analyses (PRISMA)

Positron Emitting Tomography (PET)

Random Forest (RF)

Risk of Bias (ROB)

Single Nucleotide Polymorphism (SNP)

Studies (CHARMS)

Support Vector Machines (SVM)

The Critical Appraisal and Data Extraction for Systematic Reviews of Prediction Modelling (CHARMS)

For Review Only

1 Introduction

Dementia comprises of a number of neurodegenerative disorders which cause a range of symptoms, some examples of these are memory loss, depression/anxiety and physical impairments such as incontinence[1]. The most common form of dementia is Alzheimer's disease, accounting for more than 75% of cases[2]. The main neuropathological characteristics of Alzheimer's disease are the accumulation of amyloid beta plaques and neurofibrillary tangles consisting of tau protein, which impact brain function[3].

Diagnosing the correct form of dementia has long proven difficult due to different forms sharing phenotypic characteristics[4]. Currently, the only method to confirm a diagnosis of a specific type of dementia, is postmortem brain biopsy[5]. Along with individual's age, genetics has been shown to be a strong risk factor for developing Alzheimer's disease. Twin and family studies have suggested that genetics are partially responsible for at least 80% of Alzheimer's disease cases[6]. While there are currently no specific treatments to prevent Alzheimer's disease or reverse its course, early diagnosis can enable clinicians to improve an individual's quality of life during disease progression. This can be achieved through a combination of medication and palliative care, which are most effective when commenced in an early stage of the disease. Early prediction can also provide insights to patients and caregivers, enabling them to prepare for the personal implications of Alzheimer's disease[7].

For Review Only

Machine Learning (ML) can be defined as a set of algorithms which learn underlying trends and patterns in data. It is not a novel concept, however interest in its applications has increased significantly in recent decades. This is due to modern computers being able to process larger data sets and perform in depth mathematical calculations in less time[8]. Advantages of ML lie mostly in the ability of algorithms to learn from complex datasets, with emphasis on analysing hidden relationships which may be non-linear. Therefore, ML algorithms are able to provide data-driven classifications in a multidimensional space of predictors, instead of hypothesis driven approaches testing a subset of predictors at a time[9].

Advancements in biotechnology have resulted in various aspects of human biology being reliably recorded, including genetic data and other commonly used biomarkers, e.g., cerebral

blood flow, brain imaging, etc. This has led to the accumulation of large biological data sets which ML algorithms can learn from, with the aim of classifying the participants or predict the membership of predefined classes[10]. The combination of genetic data with other data modalities often leads to complexity, which cannot be processed easily by humans in an un-biased way[11].

However, despite the advantages of using ML for answering biological questions, possible issues must be overcome in ML model development and implementation. Overfitting is a common issue when developing ML models[12], whereby a ML model does not generalise well from observed data to unseen data. In this instance, while the model may perform well when making predictions on training data, predictions are not accurate when exposed to new information. Another relevant issue which may arise when using ML is insufficient sample size. The scenario in which the number of predictors outweighs the number of samples in a dataset often leads to optimistically biased ML performance[13]. Genetic datasets are likely to fall into this category due to the many thousands of genetic markers in the human genome[14]. Therefore, a careful and clear strategy for the validation of ML models must be considered in order to prevent overfitting and overinterpretation of the results.

This review assesses the ability of ML methods to predict Alzheimer's disease using only genetic data. Initially all forms of dementia were to be examined, however searches returned publications focused on Alzheimer's disease only. The review was written in line with the Preferred Reporting Items for Systematic Reviews and Meta-analyses (PRISMA) guidelines[15]. Databases were searched for relevant scientific articles, followed by an assessment on how prediction models were developed. Reviews in this area have been conducted previously[16], however this review is unique in its assessment for the possibility of bias for prediction models in this subject area. This aspect was assessed by a specialist tool called the prediction model risk of bias assessment tool (PROBAST)[17].

2 Materials and Methods

Search Strategy

The online article databases Scopus, PubMed and Google Scholar were used to identify relevant publications for this review. Search terms used were machine learning, genetics, dementia, Alzheimer's, Single Nucleotide Polymorphism (SNP), polymorphism, mutation,

For Review Only

variant and marker. These were used to retrieve studies published between December 2009 – June 2020. An initial search and screening for relevant publications was conducted by assessing both abstracts and titles. Based on eligibility criteria (listed below), publications from the initial search were then further assessed by two independent reviewers. Any discrepancies were then resolved by a third reviewer.

Inclusion Criteria

- Written in the English language
- Subject matter of Alzheimer's disease.
- The use of SNP data
- Supervised ML techniques
- Prediction resulting in a binary outcome (i.e., case/control)

Exclusion Criteria

- Prediction of Alzheimer's disease related sub-phenotypes (e.g., MCI vs controls)
- The use of genetic material other than SNPs as predictors

For Review Only

We identified articles published between December 2009 and June 2020. ML techniques have been used in studies prior to this time frame. However, interest in ML in biological research has increased mostly in the last decade[18], therefore studies previous to this were sparse and hence this recently defined window was used. SNPs were the only form of genetic variation accepted, articles focusing on gene expression data or other forms of genetic data (e.g., rare variants, mutations) were not included. Instances where authors had combined SNP data with other forms of predictive biological variables (e.g., Magnetic Resonance Imaging (MRI) and Positron Emitting Tomography (PET) were included.

For the purpose of assessing the suitability and comparability of ML approaches, prognostic and diagnostic models were considered separately. Prognostic models are defined as those which focus on future events and use longitudinal data, whereas diagnostic models are based upon current events using cross-sectional data.

Data Extraction

The Critical Appraisal and Data Extraction for Systematic Reviews of Prediction Modelling Studies (CHARMS)[19] was used as a tool to perform data extraction. CHARMS provides two

tables of check points to be considered by the reviewer. The first table provides guidelines on how to frame the aim of a review, including how to search and filter extracted publications. The second table lists aspects which should be extracted from each study for comparison, including predictor type, sample size and the amount of missing data. CHARMS also gives guidance on assessing how certain aspects were reported such as model development, model performance and model evaluation. Advantages of using CHARMS includes replicability across different types of reviews, it's ease of use, and assisting reviewers in producing transparent publications[19].

The ability of ML methods to discriminate between two classes (cases and controls) was extracted independently from all studies by two authors. Accuracy (ACC) describes the performance of a classifier with respect to all samples, it is calculated as the number of correct predictions divided by the number of predictions made. However, it does not provide information on how well the model performs within the positive and negative classes[20]. Sensitivity is calculated by using observed positive outcomes to determine the proportion of classifications correctly made in the positive class, while specificity measures the same statistic in the negative class. Area under the receiver operating characteristic curve (AUC) represents the trade-off between these two measurements at different thresholds, aiming to find the optimal balance[20]. AUC was extracted in order to draw comparisons between the studies. Confidence intervals for AUC were also extracted if provided, otherwise these were calculated using the Newcombe method[21]. Precision can be defined as the ratio of correct predictions in the positive class, divided by the total number of positive predictions. Measures of performance such as accuracy, sensitivity, specificity and precision were also recorded alongside AUC if present. As the true positive rate and recall are different terms used for sensitivity, while specificity is also known as the true negative rate, they were categorised under sensitivity or specificity (if reported).

Statistics such as age and gender for participants, types of predictors and ML models were also extracted, as per the CHARMS checklist guidance. Also, figures in this study were created using both Microsoft Word (Fig1) and the programming language Python (Figs2&3).

Calibration is defined as a model's accuracy of predicted risk probabilities, with the goal of comparing the calculated class membership probabilities with actual observations. For example, if 60% of samples with a class membership probability of 0.6 are correctly classified,

For Review Only

then the model can be described as well calibrated. Studies were analysed in order to determine whether they reported the calibration of their models.

Data Analysis

When assessing a number of studies in a review, meta-analyses are often conducted. A meta-analysis produces a weighted average of the reported measures, where the heterogeneity between studies is taken into consideration. If studies overlap, e.g. contain (partially) the same individuals, the resulting correlation between the studies will bias the results of the meta-analysis[22], unless taken into account. Since the majority of the extracted publications used the same dataset, a meta-analysis was not performed in this review.

Risk of Bias (ROB) is another component to critically assess when conducting a systematic review of prediction models within studies. PROBAST uses a system of questions split over four categories: participants, predictors, outcome and analysis. Each category contains multiple choice questions assessing an occurrence of shortcomings in that category (with choice of answers from: “yes”, “probably yes”, “no”, “probably no” and “no information”). If any question is answered with no or probably no, this flags the potential for the presence of bias, however assessors must use their own judgement to determine whether a domain is at ROB or not. An answer of no does not automatically result in a high ROB rating. PROBAST does offer assistance on how to reach an overall conclusion on the level of bias in that category. In this review we assessed all selected studies for ROB.

For Review Only

3 Results

3.1 Search Results

Following an initial search, a total of 4020 publications were returned. This number was reduced by assessing whether both titles and abstracts aligned with inclusion criteria, resulting in 500 studies. A more in-depth analysis was then conducted on the full texts, removing publications which did not pass inclusion criteria upon a detailed inspection, 25 texts passed this stage. These were further reduced to 21 due to the presence of duplicates, comprising of both pre-prints and conference abstracts. Nine further publications were then removed due to non-relevant methodologies, leaving a final set of 12 studies to be included. A visual representation of the selection process is given in Fig 1.

Fig 1. Visual breakdown of publication selection based on a similar diagram found in PRISMA.

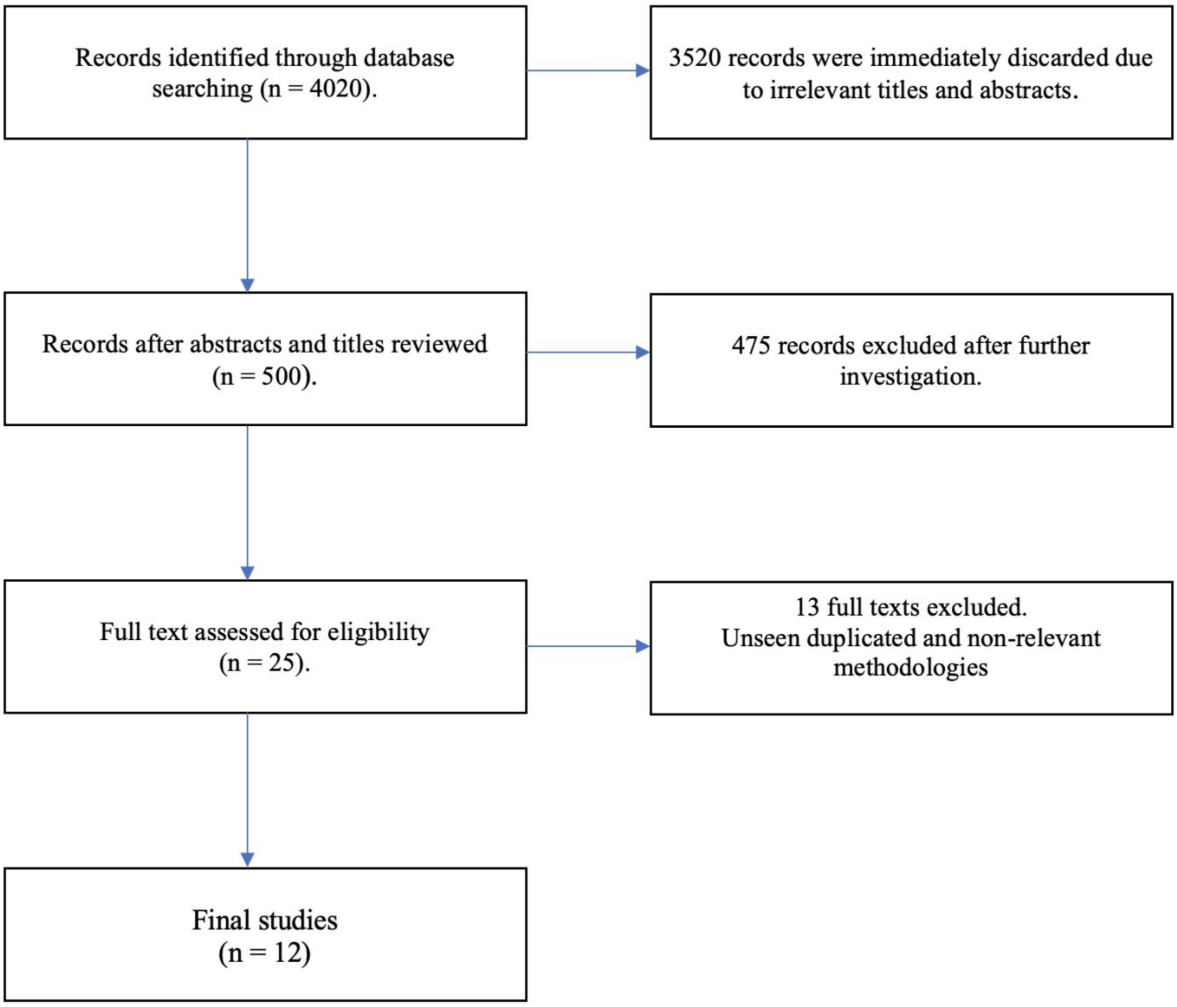

The majority of publications (10/12) used the publicly available Alzheimer's Disease Neuroimaging (ADNI)[23] data set. ADNI is a longitudinal study measuring various biomarkers in both Alzheimer's disease cases and healthy age-matched controls. However, all studies reported here analysed a particular subset of the cohort at a fixed timepoint only. Therefore, models used throughout these publications were classed as diagnostic (not prognostic). There were two studies that did not use ADNI. Wei, Visweswaran and Cooper [24] used a combination of three data sets[25] in which biomarkers were collected at a fixed time point, therefore data were cross-sectional. Romero-Rosales *et al.*[26] used a longitudinal source of data known as the National Institute on Aging-Late-Onset Alzheimer's Disease Family Study (NIA-LOAD)[27].

Again, values for predictors were taken at a fixed time point, thus the data used was cross-sectional. All models across included studies were classified as diagnostic.

A range of machine learning approaches were used across the 12 reviewed studies. Table 1 outlines all types of models used and their frequency across publications. The most commonly used ML approach across publications was Support Vector Machines (SVM), followed by Naïve Bayes (NB) and Penalised regression. The number of tested models was also the highest for SVMs. This approach allows the most flexibility when adapting models via kernel functions[28]. Penalised regression was commonly used in the form of the Least Absolute Shrinkage and Selection Operator (LASSO). This type of regularisation shrinks coefficients closer to zero when compared to their maximum likelihood estimates, and so simultaneously reduces variance in predictions and performs predictor selection. These aspects make penalised regression a popular method in prediction analysis[29]. RFs were also used across three studies, these algorithms are intuitive in their use of decision trees, are invariant to scaling, and provide an in-built measure of predictor importance, which likely explains their favour in biology[30]. Supplemental Table 1 outlines how the model types displayed in Table 1 were distributed across publications. It also provides study names; sample sizes and which methods were used to report results. The most commonly used statistics for model performance were ACC and AUC. With five studies reporting AUC and the remaining seven studies reporting ACC.

Table 1. Summary of ML methods used in publications.

ML approach[a]	Number of publications[b]	Number of models reported across publications[c]	Additional Information[d]
Support Vector Machine (SVMs)	8	44	Linear kernels (22 models, 5 studies). Quadratic polynomials (4 models, 2 study). Cubic Polynomials (4 models, 2 study). Radial basis functions (3 models, 2 studies). Pearson kernel function (2 models, 1 study). Unreported kernels (9 models, 3 studies). A supervised method which uses distance-based calculations to separate samples into groups.
Penalised Regression (LASSO)	4	15	All 15 LASSO regressions across 3 studies. A regression analysis which performs both feature selection and regularisation.
Naïve Bayes (NB)	4	10	Six ordinary NB models, three tree-augmented NB and one model averaged NB. A probabilistic

For Review Only

			classifier which uses bayes theorem to make predictions.
Random Forest (RF)	3	5	Five classification RFs used, two of which used the RPART package. These are an ensemble of decision trees which produce aggregated classifications.
Bayesian Networks (BN)	2	4	2 BNs with K2 learning algorithm, one markov blanket and one minimal augmented markov blanket. A graphical model which calculates conditional dependencies between variables using Bayesian statistics.
Linear Models	2	4	Bootstrapping Stage-Wise Model Selection (BSWiMS). A supervised model-selection algorithm which uses a combination of linear models for prediction.
K Nearest Neighbour (KNN)	2	3	This is a distanced based algorithm which uses similarities in features to classify.
Ensemble Methods	1	2	Ensembles are the use of a number of ML models, these arrive at a collective prediction result.
Logistic Regression (LR)	1	1	A form of linear regression whereby the outcome is a categorical variable.
Multi-Factor Dimensionality Reduction (MFDR)	1	1	A technique used to detect combinations of independent variables that influence a dependent variable.

Random Forest (RF), Bayesian Networks (BN), K Nearest Neighbour (KNN), Logistic Regression (LR), Multi-Factor Dimensionality Reduction (MFDR)

3.2 Risk of Bias (ROB)

For diagnostic models, data sources with the lowest risk of ROB for participants are of the cross-sectional form. The publications which used the ADNI data set assessed it in a cross-sectional format. This assertion is reinforced in Gross *et al.*[31], where ADNI is described as a cross-sectional study with longitudinal follow up. A similar decision was reached when considering the two studies which did not use ADNI, Wei, Visweswaran and Cooper[24] and Romero-Rosales *et al.*[26] . After considering this, ROB was deemed low for participants.

The focus of PROBAST for predictors is to assist the reviewer in determining whether the procedures for measuring biomarkers were equal for all members of the study. ADNI provides publicly available documents which outline the methods for biomarker collection. Predictors derived from blood samples or MRI scans were collected using the same protocols for all participants. However, time intervals between measurements differed for Alzheimer's disease patients and those deemed either controls or MCI study participants. Alzheimer's disease patients were seen at less regular intervals than MCI individuals. Despite being seen more

For Review Only

regularly than Alzheimer's disease patients, the protocols used for each MCI assessment did not differ between data collection points. Also, the more frequent collection of biomarkers for MCI individuals did not affect assessments for both Alzheimer's disease and control study members. Therefore, this time interval difference was considered to be of low ROB. Genotyping of SNPs for the NIA-LOAD data set[27] was uniform across all samples, therefore ROB for predictors was low for Romero-Rosales *et al.*[26] . Procedures for collecting predictors in Wei, Visweswaran and Cooper[24] were not provided. This was also the case when assessing the original source of the data by Romero-Rosales *et al*[26] therefore ROB for predictors for these publications was stated as not known.

Blinding is the process whereby samples from patients are collected without prior knowledge of their disease status. Such knowledge has been shown to introduce bias to collection procedures[32]. According to the ADNI data generation policy, samples were collected using blinding and only unblinded when uploaded to databases. Imaging data were collected and processed using standardised automated pipelines, thereby reducing the possibility of multiple clinicians using different methods when collecting predictors[33]. ROB was deemed low for blinding in ADNI. Policies for blinding were not provided by either Wei, Visweswaran and Cooper[24] or Romero-Rosales *et al.*[26]. Therefore, a judgement could not be made for either publication.

ROB in the PROBAST category "outcome" was considered to be low for the majority of studies. PROBAST's questions regarding this section focus on how the outcome was determined and whether this determination was applied equally to all participants. ADNI used a range of clinically accepted methods to determine an individual's Alzheimer's disease status, including the Mini Mental State Examination and the Clinical Dementia Rating. The use of multiple methods of cognitive performance reduced the possibility of misdiagnosis, which in turn reduced the ROB. Diagnosing the outcome for participants in NIA-LOAD study was also achieved using a range of stringent methods. NINCD-S-ADRDA[34] criteria were used for Alzheimer's disease diagnosis at recruitment, while diagnosis was pathologically confirmed for participants who were deceased. Controls were determined using neuropsychological tests in which memory function was examined, coupled with examination for any previous history of neurological disorders. As methods for both controls and cases were applied uniformly across the study participants, with the exception of deceased and alive Alzheimer's disease individuals, the ROB for Romero-Rosales *et al.*[26] was deemed low for outcome. In Wei,

For Review Only

Visweswaran and Cooper[24] all brain donors for cases satisfied clinical and neurobiological criteria for cases of late onset Alzheimer's disease, while clinical cases satisfied criteria for probable Alzheimer's disease[35]. Also, brain donor controls did not have significant cognitive impairment at the time of death and clinical controls exhibited no cognitive impairment. However, the methods used to determine these diagnoses were not elaborated upon. For instance, whilst there was a mention of using clinical criteria, these were not defined. Therefore, ROB for outcome was unclear.

The fourth and final category in which PROBAST aids investigation is in the analysis phase of a study. All studies exhibited high ROB for this section, with a consistent lack of reporting for calibration; additionally, 5 out of 12 publications did not report possible missing values in their data and how these were dealt with if present. To assess whether sample sizes used in modelling are adequate, PROBAST suggests the use of the metric Events per Variable (EPV). EPV is defined as the number of events in the minority class (i.e., the smaller of either cases or controls), divided by the number of candidate predictors used. In cases where more in depth algorithms (e.g., Neural Networks (NNs)) are used, model parameters are also included in the calculation of EPV. We evaluated ROB using a value of at least 10 EPVs, following common recommendations [13]. However, this threshold maybe tailored more to the accurate estimation of regression coefficients in a logistic regression model. More complex algorithms which require the tuning of hyperparameters (RFs, SVMs, NNs) may require a value of over 100[36]. Values across all studies were assessed to be below this threshold. The study with the highest EPV of 9.43 was Chang *et al.*[37]. The lowest EPV, 0.0018, was found for Wei, Visweswaran and Cooper[24].

Values of EPV below the recommended threshold of 10 introduce the possibility of overfitting, which in turn may result in spurious results[13]. However, efforts were made by most studies to overcome the problem of overfitting, mostly in the form of Cross-Validation (CV) (11/12 studies). During this process, the data is divided into *k* partitions, with *k*-1 partitions used as training data and the remaining partition used as the test set. This process is then repeated *k* times. It has been demonstrated that using CV is a viable method for authors to address overfitting[38]. Despite this, the possibility of bias could still be present if the correct form of CV is not chosen. To investigate the importance of CV type selection, several methods of CV were used on data sets with low EPV values[39]. The simplest form of CV (*k*-partitioning) was shown not to counteract the issue of overfitting in some instances and could even exacerbate the

For Review Only

problem. Nested-CV was shown to achieve the best performance of all methods[40], this method operates by using an outer and inner loop of CV. The outer loop splits *k* times to perform model validation while hyperparameters and feature selection are conducted in the inner loop. This method was only reported by one of the included studies[41].

3.3 Machine Learning Performance

Figures 2 and 3 summarise the reported accuracies across all studies and ML methods. The first column shows the reference number of the publication as listed in Supplemental Table 1, along with the sample size used in in the respective ML model. ML approaches used are shown in the second column. The third column displays information which assists the reader in distinguishing between models in the same study, this includes such factors as number of SNPs used and differing methodologies implemented. Studies were sorted by sample size in ascending order. The vertical dashed line shows the accuracy of 0.5, which indicates a 50% chance of the result being correct (the theoretical lowest value of AUC or ACC). The last column shows the actual values of the accuracy achieved.

For Review Only

Five studies recorded AUC for the performance of models, ranging from 0.49 to 0.97. The remaining seven studies reported mainly ACC, sensitivity and specificity (Supplementary Table 2). The highest AUC value was achieved by An *et al.*[42] (Study 6 in Figure 1), where authors used a hierarchal method to find the optimal set of features for the prediction of Alzheimer's disease. Manifold regularisation was used to combine both genetic and MRI data in a semi-supervised hierarchal feature and sample selection framework. This method utilised both labelled and unlabelled data in order to maximise the amount of information for prediction. For classification purposes, SVMs were used to discriminate between controls and cases. However, the EPV score was 0.919, this is below the recommended threshold of 10. This could introduce the possibility of overfitting which can in turn lead to spurious results[13]. The authors used CV to in order to alleviate the potential for overfitting.

A single study reported calibration statistics[24] (Publication 5 in Supplementary Table 1). Authors compared the predictive capability of a model using averaged NB with both standard NB and NB with feature selection. The method used to report calibration was calibration curves. Results highlighted that the model using averaged NB achieved better calibration than

the standard NB model, and offered similar performance to the NB with feature selection. The prediction accuracy of these models was 0.59-0.72 (Publication 5 in Figure 1).

Ten-fold CV was the most common form of validation used, however a range of other values of *k* were also documented. One further study used a nested CV approach to optimise both model performance and hyperparameter tuning. Leave one out CV was also used by a singular study, this functions by creating a number of folds equal to the number of data points in the training set. Within each fold a single data point is removed to be used as the test set, the algorithm is then trained on the remaining points. Prediction performance is calculated by averaging over the results for all folds. Also, one publication explored a different approach of dividing the data into training and test datasets called a split sample. In this process, a model is trained using a training set and is subsequently tested on a validation (test) set. Where the test dataset comprises of the remainder of the original data not included in the training dataset. All of these methods are known as internal validation, where model optimisation and hyperparameter tuning is achieved using a singular data set. External validation involves using a completely separate cohort to validate an already trained model, usually this cohort has been independently gathered and assessed to the initial training data[43]. This method was not used by any study in this review (Supplementary Table 3).

For Review Only

Fig 2. A forest plot displaying models used across publications which reported AUC, with the addition of confidence intervals derive using the Newcombe Method.

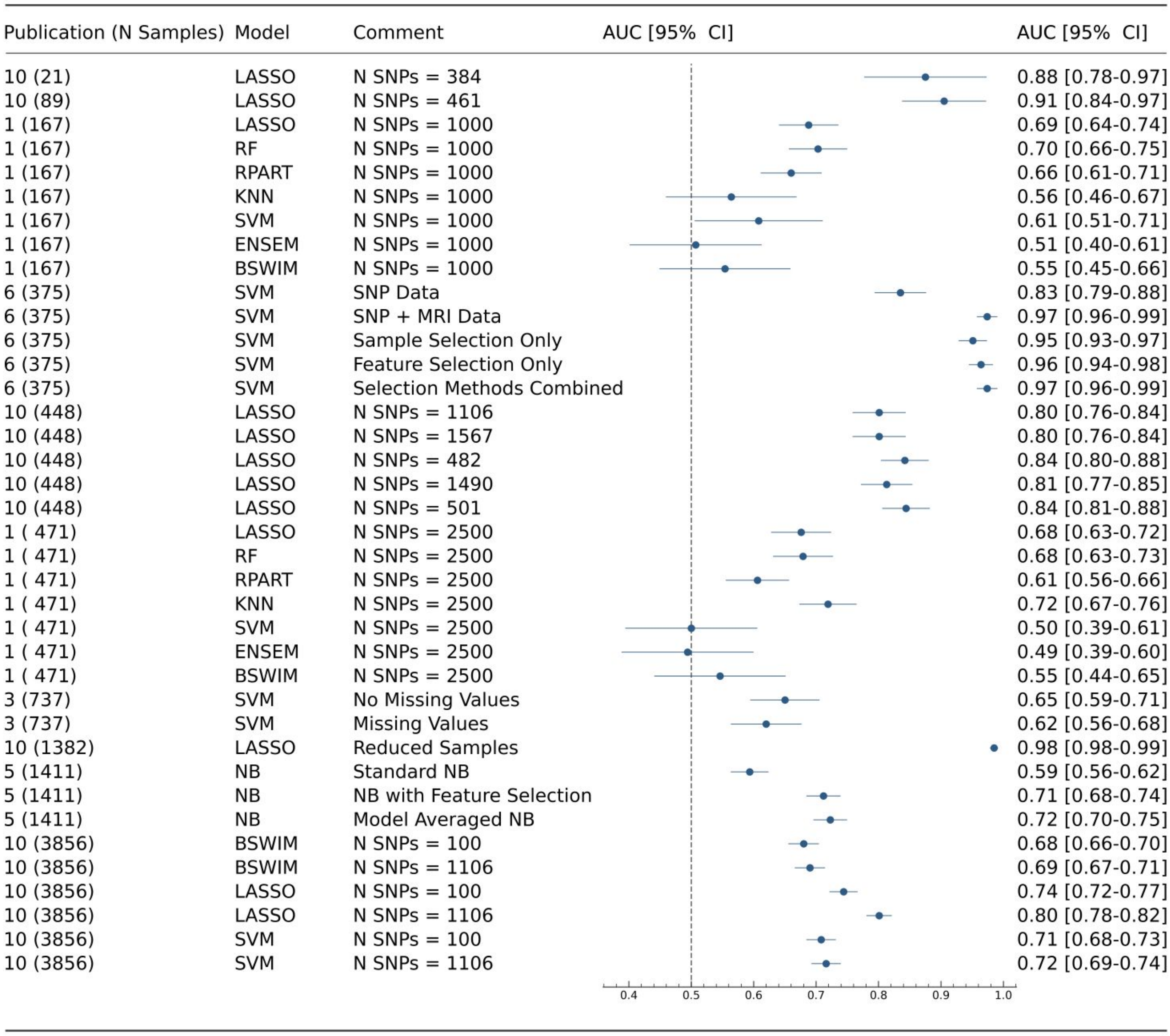

Fig 3. A forest plot displaying all models used across publications which reported ACC.

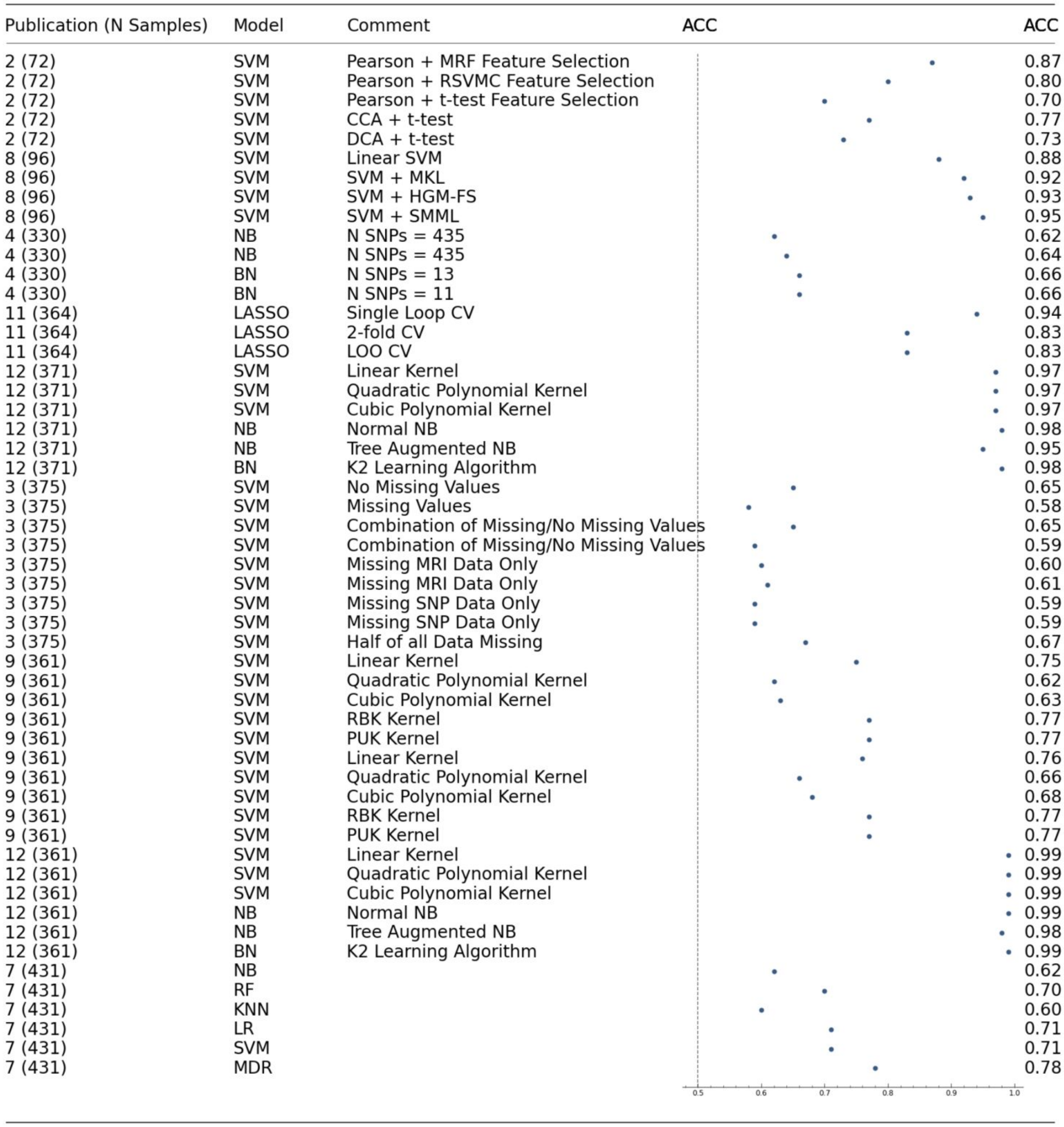

3.4 Sample Size

Sample sizes ranged from 72 to 3,856 individuals, with the largest cohort being the NIA-LOAD data set[44]. The majority (10/12) of studies used 300-900 individuals from the ADNI data set. The number of SNPs used in models varied between studies, with numbers ranging from 21 to 561,309 SNPs. The large range in the number of SNPs used was due to differences in methodologies. The study which used the greatest number of SNPs[26] investigated improving AUC by reintroducing initially misclassified samples to the final models. The study which used the least number of SNPs focused only on the top 10 genes associated with Alzheimer's disease[45], thereby limiting the number of SNPs included in the study. EPV ranged from 0.0018-9.43 for eleven studies, with one study not providing enough information to calculate EPV. Imbalances between classes, as a ratio between controls over cases, ranged from 0.408-6.55, with a median value of 1.193 (Supplementary Table 4). The accuracy for the study with the highest class imbalance (6.55) was 0.95-0.99 ACC[46].

3.5 Predictors

Criteria used for inclusion specified that SNPs were the only form of genetic data used as predictors. However, other predictors were also considered, whereby other forms of predictive material were used alongside SNPs. The most common form of secondary data used was MRI, included in four publications. PET imaging data was also used in two studies. Additionally, Cerebrospinal Fluid (CSF) was used in one publication (Supplementary Table 5).

Pre-processing techniques for SNPs were reported in the majority (8/12) of studies. All of these studies excluded SNPs which did not satisfy Hardy-Weinberg equilibrium[47]. SNPs were selected with a variety of Alzheimer's disease association significance thresholds (0.00007 – 0.05), leading to differing numbers of SNPs being retained across studies. Seven of the studies which discussed pre-processing for SNPs also reported the minimal Minor Allele Frequency (MAF). Rare variants were removed from a SNP set as they occur only in a small number of individuals in a population. Thresholds used for MAF varied (0.01 – 0.04) across studies (Supplementary Table 5.) Four studies did not report steps taken to pre-process SNPs; this could lead to questions regarding data quality.

For Review Only

Eight out of 12 studies used methods to address missing data values. Two studies excluded samples with missing predictor values above or equal to a threshold of 10%. A further four publications described processes for the imputation of missing genotypes. For instance, Sherif, Zayed and Fakhr[48] imputed missing SNP values by the use of the expectation maximisation algorithm. Another study[26] imputed missing genotypes by using the median value of the nearest neighbours, this was the only example of using a measure of central tendency. Zhou *et al.*[41] did not remove or impute missing data, rather they designed a method in which samples with missing values were incorporated in the models. All complete samples were used to develop a latent representation space. Samples with missing values were used to learn independent modality specific latent specifications. These latent representations were then used as an input for the Alzheimer's disease classifier. This process allowed these authors to produce models which outperformed comparable methods of dealing with missing data and selecting features.

None of the studies which reported the use of imputation methods specified whether this process was undertaken before CV or afterwards, which may be prone to the issue of data leakage [49].

3.5 Hyperparameter search

Hyperparameter tuning is a common step in developing prediction models, it is implemented to ensure the optimisation of AUC[50]. Reporting of techniques for hyperparameter optimisation was inconsistent across studies, with seven publications not providing values or the process of tuning. For the remaining five studies, a range of differing techniques were used. Zhou *et al.*[41] used a nested approach to optimise model parameters. Ten-fold CV was used to fit models, whilst an inner loop of five-fold CV trained model hyperparameters. However, this was only the case for some hyperparameters, as some were fixed at pre-determined values to reduce training times. This arbitrary fixing of values may have introduced bias. This study was the only one to report the use of nested CV for hyperparameter tuning.

Further studies described the use of CV for hyperparameter optimisation, for instance, Bi,Cai, Wang, and Lui[51] used an iterative process to determine the optimum number of decision trees to use in their RF approach. Furthermore, grid search and CV techniques were employed to optimise varying hyperparameters across the other five studies (Supplementary Table 6). In this process, CV is used to test different combinations of hyperparameter values, with the aim

For Review Only

of producing the set which leads to the highest value of AUC. Seven publications did not report optimisation methods. Of these seven studies, four used NB methods, which do not require hyperparameter tuning. For the remaining three studies, hyperparameter tuning was required but not reported.

3.6 Descriptive statistics.

Nine studies did not report values regarding both age and gender for study participants. The remaining three reported the age and gender distributions in both classes (cases and controls). De Velasco Oriol *et al.*[52] reported age and gender for both the discovery and validation sets. Values for the mean age for both cases (75.4-75.5) and controls (76.1-77.4) were similar across studies. This similarity is due to the consistent use of the ADNI dataset throughout studies. The proportion of males to females' controls ranged from 0.59-1.22; in cases, this proportion ranged from 1.05-1.22(Supplementary Table 7).

4 Discussion

This review assessed a selection of studies which used ML to predict Alzheimer's disease from mainly genetic data. Using a systematic approach (PRISMA), 12 studies were identified which met the inclusion criteria. These manuscripts were reviewed to identify the type of models used, model development and the validity of the reported results.

AUC results in the included studies (5 out of 12) varied (0.49-0.97) for Alzheimer's disease risk prediction. The most accurate models were shared across two studies, with authors recording AUC of 0.8 or above. In any field, these results would be deemed as impressive. More specifically to the topic of Alzheimer's disease prediction, these results match and, in some instances, outperform the use of Polygenic Risk Score which has been shown to achieve AUC of 75-84% across a range of studies outlined in a review[53]. Seven out of 12 publications did not report AUC for their models, with accuracy, sensitivity and sensitivity being the preferred choices. The most common measure of performance used other than AUC was ACC. Four studies reported ACC of above 0.8, which again can be considered important when attempting to reduce the possibility of miss-communicated risks to individuals. However, ACC can be skewed by the presence of class imbalances, which have been reported in this paper[54]. In addition, ACC is calculated from all predictions against all observed outcomes; this does

For Review Only

not clarify how the model performs per class. For these reasons we advocate that AUC should be used as a standard measure for reporting performance.

Continued research and development in the field of ML has led to an increasing number of algorithms available for use in prediction[55]. This is reflected in the usage of 10 different types of approaches across all studies, the most popular of these being Support Vector Machines. SVMs are known for their simple application and predictive accuracy, and are therefore used regularly in prediction modelling[56]. Other notable algorithms used in the assessed studies were Random Forests (RFs) and Naïve Bayes (NB). Similar to SVMs, NB is known for its easy implementation. However, its performance can be hindered due to correlations between features, which negates the naïve assumption that all input features are independent[57]. If correlation between features is present, the importance of these features will be overemphasised during modelling[58]. Random Forests (RFs), used in three studies, are a popular form of classifier due to their ability to negate overfitting. However, applying RFs to prediction problems can be challenging due to the need for hyperparameter tuning[59]. Given the success of the forementioned algorithms in a range of application areas, it is perhaps not surprising these three algorithms were the most used across all publications[60].

None of the included studies used Neural Networks (NNs) to predict Alzheimer's disease. NNs are powerful predictive algorithms, with the ability to learn non-linear patterns in complex data sets. In some scenarios, they can infer relationships in data which are beyond the scope of other ML techniques[61]. A possible explanation for their absence could be the structure of data sets used across the selected models, where the number of predictors often outnumbered individuals. The scenario in which a data set has many more predictors than individuals can lead to overfitting[62]. NNs are known for being complex to implement, as well as difficult for hyperparameter tuning and susceptible to overfitting[63] . This may explain why they were not used in the reviewed studies.

A number of consistent issues were highlighted across the included studies. One of the main focus points was the widespread usage of the ADNI data set, with 11 of the 12 included studies using this as a data source. Methods used to demonstrate model performance were reported inconsistently. The combination of low EPV values and inconsistent model performance reporting led to the possibility of bias in the analysis phase of modelling.

For Review Only

In terms of model implementation, the main aspects scrutinised were the use of any hyperparameter tuning, as well as the methods used for model validation. Hyperparameter tuning has become an increasingly important part of ML development. The majority of algorithms require certain values for hyperparameters which are specified by the user. If these values are not optimised, then the model is susceptible to overfitting and inaccurate predictions[64] . Five out of the 12 studies referenced the use of hyperparameters, the remaining 7 studies did not outline any tuning methods. Greater transparency about the use of hyperparameters and their tuning allows the reader to understand whether issues such as overfitting were accounted for. Therefore, researchers should report both hyperparameter values and methods used to obtain them.

Model validation is also an important aspect of predictive analysis. Correct methods of validation reduce the likelihood of overfitting, whereby algorithms become too reliant on the data trained upon and cannot perform sufficiently when tested on unseen data[39]. The most commonly used method among the selected studies (11/12) was CV. This method has become increasingly popular in prediction models, due to its ability to counteract overfitting[65]. Eleven of the 12 studies which reported CV used a varying number of folds, the remaining publication used a technique called Leave one out CV. In the majority of cases, the higher the number of folds, the greater the accuracy from CV. However, increasing the number of folds leads to a higher chance of overfitting. Therefore, Leave one out CV is only suitable for small data sets, where the number of samples is 100 or less[66]. Nested CV was used by one study only[67]. This was the only evidence of using separate validation folds for both model optimisation and hyperparameter tuning throughout all included studies. Using the same CV split for both of these tasks can introduce overfitting[40], therefore we recommend the use of nested CV for future analysis. The only publication which did not report CV used a train and test split method for internal validation. The model is trained only once, increasing the chance of a model becoming too reliant on the training data and thereby reducing its ability to replicate in independent datasets. Since the split of the data is conducted randomly, an argument could be made that any results derived could be influenced by this single split[68]. Therefore, methods which use a form of CV are recommended.

Calibration compares the similarity of probabilistic predictions with observed outcomes. This metric was only reported in one study[24]. Calibration is of high importance when assessing ML performance, this is especially true when considering models which may be implemented in

For Review Only

the medical sector[69]. The implications of incorrectly communicating the risk of developing Alzheimer’s disease to an individual could cause considerable harm, by means of both physical and psychological trauma. With the potential of causing death due to incorrect treatment in the most serious of circumstances[70]. Therefore, we recommend that authors aim to produce highly calibrated models and also report calibration statistics.

Another aspect investigated was sample sizes used in the training of models. These were relatively small with most studies using between 300-900 individuals (due to the common usage of the ADNI dataset). Differing methodologies also caused the number of predictors (SNPs) to vary across publications, ranging between tens of SNPs to over 100,000. The combination of small samples and large quantities of predictors led to low EPV scores, the highest of which was 9.43 Chang *et al.*[37]. The common use of ADNI also led to low EPV values due to the consistent implementation of small numbers of participants and high numbers of predictors, which may lead to model overfitting[71]. Therefore, all scores were below the threshold recommended by PROBAST. Small sample size may be a difficult issue to overcome, as genetic data is not always readily available. In this instance, it is advisable to use CV to reduce the impact of possible overfitting. However, using the simplest form of CV can in some circumstances increase the level of overfitting. Further techniques, such as nested CV have been shown to mitigate overfitting more effectively[39]. We therefore encourage authors to investigate which type of validation technique would be suitable for their models.

Class imbalances can lead to poorer prediction due to the model favouring the majority class. Techniques such as under/over sampling can be used in order to overcome this issue. Between the two methods, under sampling has been found to be more effective in addressing predictive bias[72]. This is due to a common issue amongst over sampling algorithms, in which the creation of synthetic minority samples can introduce noise to the data[73]. The issue of class imbalance was not of major concern in the reviewed papers, however with the availability of large population cohorts (e.g., UK Biobank), care should be taken when analysing diseases with small prevalence (which includes Alzheimer’s disease and other dementias).

Data leakage is another issue to be discussed. It occurs when an algorithm’s performance is artificially inflated due to information being leaked from the training to test dataset. Manipulating data before training and validation may inadvertently leak information and boost

For Review Only

performance. A way in which this can occur is pre-processing on the entire dataset before data is split. This is relevant to missing values imputation, derivation of and adjustment for population structure. In order to avoid this, any pre-processing steps should be carried out separately in both the training and test data sets[49]. To achieve non-biased results, a ML algorithm should always be validated on data separate to training data. Nested CV can be used to ensure pre-processing is carried out per fold, this reduces the risk of data leakage[74].

ROB in the remaining three sections of PROBAST (participants, predictors and outcome) was considered to be low for all publications. The usage of cross-sectional data reduced the ROB for the study participants. The use of a well-documented dataset (ADNI) provided details in areas such as predictor collection, the determination of disease status and inclusion of individuals in these studies. These areas could not be assessed in the two studies which did not use ADNI. The widespread use of ADNI also provided the possibility of comparison between studies due to the common data samples, however this prevented the possibility of performing a meta-analysis. Usage of a greater range of data sources in future studies would be beneficial for the development of ML models to improve their robustness and replicability. In particular continued usage of the same resource does not provide insight into the performance of ML in different cohorts. If used in frontline medicine, models will have to be able to predict upon individuals from different backgrounds[75]. For instance, 93% of the participants of ADNI were Caucasian[23]. It has been shown that genome wide association studies results from primarily Caucasian subjects are not applicable to other races, which may also impact the prediction success of ML algorithms trained on them[76]. Overall, despite ROB being low for the first three sections of PROBAST, issues within the analysis phase of modelling introduced concerning possibilities of bias. This could bring the validity of results into question.

This review has highlighted a number of areas which require improvement in the field of ML for disease prediction using genetic data. Some areas require greater attention than others, namely the reporting of model performance and model development. Reporting these measures thoroughly will allow for an accurate comparison between studies and provide better clarity for the performance of the models. More detailed description is also required when explaining model implementation, with special emphasis on hyperparameter tuning. This will provide greater understanding of how authors have attempted to maximise performance and reduce the possibility of overfitting. Furthermore, the majority of studies in this review used the publicly available ADNI data set, which demonstrated a clear overreliance on one particular data source.

For Review Only

Using a more diverse range of data sources would enhance the validity of results and also develop understanding of the applications of ML for Alzheimer's disease prediction. However, it is accepted that barriers can exist when attempting to access data resources, due to factors such as patient confidentiality, time taken to grant access and restriction on use[77]. ML will continue to be used more extensively in both the research and industry sectors due to its ability to analyse complex patterns in datasets, which will allow users to achieve feats not possible with more classical statistical methods[78]. Therefore, an unbiased, consistent and unified way of reporting model implementation and results is required in order to provide a substantial benefit for the advancements of ML for disease risk prediction from genetic data.

5 Acknowledgments

All contributors to this research have been named as authors

For Review Only

6 Funding

We thank the Dementia Research Institute [UKDRI supported by the Medical Research Council (UKDRI-3003), Alzheimer's Research UK, and Alzheimer's Society], Joint Programming for Neurodegeneration (MRC: MR/T04604X/1), Dementia Platforms UK (MRC: MR/L023784/2), MRC Centre for Neuropsychiatric Genetics and Genomics (MR/L010305/1), Wellcome Trust (IK PhD studentship) and the European Regional Development Fund, through the Welsh Government.

7 Competing Interests

The authors report no competing interests.

<u>Figure Legends</u>

Fig1: **Visual breakdown of publication selection based on a similar diagram found in PRISMA.**

Fig2: **A forest plot displaying models used across publications which reported AUC, with the addition of confidence intervals derive using the Newcombe Method.**

Column1 – Publication number as found in Supplementary Table 1, along with sample size. Column2 – Type of machine learning model. Column3 – Information to help distinguish between models in publications, including differing SNP numbers and methodologies.

Fig3: **A forest plot displaying all models used across publications which reported ACC.**

Column1 – Publication number as found in Supplementary Table 1, along with sample size. Column2 – Type of machine learning model. Column3 – Information to help distinguish between models in publications, including differing SNP numbers and methodologies.

Table 1. Summary of ML methods used in publications.

a – Type of machine learning model; b – The number of publications models were used in; c – The number of publications these models occurred in.

For Review Only

1. Bature F, Guinn B-A, Pang D, Pappas Y. Signs and symptoms preceding the diagnosis of Alzheimer's disease: a systematic scoping review of literature from 1937 to 2016. *BMJ Open*. 2017;7(8):e015746. doi:10.1136/bmjopen-2016-015746

2. Duong S, Patel T, Chang F. Dementia: What pharmacists need to know. *Can Pharm J Rev Pharm Can*. 2017;150(2):118-129. doi:10.1177/1715163517690745

3. Schachter AS, Davis KL. Alzheimer's disease. *Dialogues Clin Neurosci*. 2000;2(2):91-100.

4. Karantzoulis S, Galvin JE. Distinguishing Alzheimer's disease from other major forms of dementia. *Expert Rev Neurother*. 2011;11(11):1579-1591. doi:10.1586/ern.11.155

5. Paraskevaidi M, Martin-Hirsch PL, Martin FL. Progress and Challenges in the Diagnosis of Dementia: A Critical Review. *ACS Chem Neurosci*. 2018;9(3):446-461. doi:10.1021/acschemneuro.8b00007

6. Tanzi RE. The genetics of Alzheimer disease. *Cold Spring Harb Perspect Med*. 2012;2(10). doi:10.1101/cshperspect.a006296

7. Solomon PR, Murphy CA. Early diagnosis and treatment of Alzheimer's disease. *Expert Rev Neurother*. 2008;8(5):769-780. doi:10.1586/14737175.8.5.769

8. Attaran M, Deb P. Machine Learning: The New "Big Thing" for Competitive Advantage. *Int J Knowl Eng Data Min*. 2018;5(1):1. doi:10.1504/IJKEDM.2018.10015621

9. Sidey-Gibbons JAM, Sidey-Gibbons CJ. Machine learning in medicine: a practical introduction. *BMC Med Res Methodol*. 2019;19(1):64. doi:10.1186/s12874-019-0681-4

10. Cho G, Yim J, Choi Y, Ko J, Lee S-H. Review of Machine Learning Algorithms for Diagnosing Mental Illness. *Psychiatry Investig*. 2019;16(4):262-269. doi:10.30773/pi.2018.12.21.2

11. Sivarajah U, Kamal MM, Irani Z, Weerakkody V. Critical analysis of Big Data challenges and analytical methods. *J Bus Res*. 2017;70:263-286. doi:10.1016/j.jbusres.2016.08.001

12. Yeom S, Giacomelli I, Menaged A, Fredrikson M, Jha S. Overfitting, robustness, and malicious algorithms: A study of potential causes of privacy risk in machine learning. *J Comput Secur*. 2020;28(1):35-70. doi:10.3233/JCS-191362

13. Austin PC, Steyerberg EW. Events per variable (EPV) and the relative performance of different strategies for estimating the out-of-sample validity of logistic regression models. *Stat Methods Med Res*. 2017;26(2):796-808. doi:10.1177/0962280214558972

14. Mei B, Wang Z. An efficient method to handle the "large p, small n" problem for genomewide association studies using Haseman-Elston regression. *J Genet*. 2016;95(4):847-852. doi:10.1007/s12041-016-0705-3

15. Liberati A, Altman A, Tetslaff J, Moher D. The PRISMA Statement for Reporting Systematic Reviews and Meta-Analyses of Studies That Evaluate Health Care Interventions: Explanation and Elaboration. Published online August 2009.

16. Mishra R, Li B. The Application of Artificial Intelligence in the Genetic Study of Alzheimer's Disease. *Aging Dis*. 2020;11(6):1567-1584. doi:10.14336/AD.2020.0312

17. Wolff RF, Moons KGM, Riley RD, et al. PROBAST: A Tool to Assess the Risk of Bias and Applicability of Prediction Model Studies. *Ann Intern Med*. 2019;170(1):51. doi:10.7326/M18-1376

18. Camacho DM, Collins KM, Powers RK, Costello JC, Collins JJ. Next-Generation Machine Learning for Biological Networks. *Cell*. 2018;173(7):1581-1592. doi:10.1016/j.cell.2018.05.015

19. Moons KGM, de Groot JAH, Bouwmeester W, et al. Critical appraisal and data extraction for systematic reviews of prediction modelling studies: the CHARMS checklist. *PLoS Med*. 2014;11(10):e1001744. doi:10.1371/journal.pmed.1001744

20. Flach P. Performance Evaluation in Machine Learning: The Good, the Bad, the Ugly, and the Way Forward. *Proc AAAI Conf Artif Intell*. 2019;33:9808-9814. doi:10.1609/aaai.v33i01.33019808

For Review Only

21. Debray TP, Damen JA, Riley RD, et al. A framework for meta-analysis of prediction model studies with binary and time-to-event outcomes. *Stat Methods Med Res*. 2019;28(9):2768-2786. doi:10.1177/0962280218785504

22. Bom PRD, Rachinger H. A GENERALIZED-WEIGHTS solution to sample overlap in META-ANALYSIS. *Res Synth Methods*. 2020;11(6):812-832. doi:10.1002/jrsm.1441

23. Petersen RC, Aisen PS, Beckett LA, et al. Alzheimer's Disease Neuroimaging Initiative (ADNI): clinical characterization. *Neurology*. 2010;74(3):201-209. doi:10.1212/WNL.0b013e3181cb3e25

24. Wei W, Visweswaran S, Cooper G. The application of naive Bayes model averaging to predict Alzheimer's disease from genome-wide data. *Natl Libr Med*. Published online July 2011.

25. Reiman EM, Webster JA, Myers AJ, et al. GAB2 alleles modify Alzheimer's risk in APOE epsilon4 carriers. *Neuron*. 2007;54(5):713-720. doi:10.1016/j.neuron.2007.05.022

26. Romero-Rosales B-L, Tamez-Pena J-G, Nicolini H, Moreno-Treviño M-G, Trevino V. Improving predictive models for Alzheimer's disease using GWAS data by incorporating misclassified samples modeling. Gwak J, ed. *PLOS ONE*. 2020;15(4):e0232103. doi:10.1371/journal.pone.0232103

27. Lee JH, Cheng R, Graff-Radford N, Foroud T, Mayeux R, National Institute on Aging Late-Onset Alzheimer's Disease Family Study Group. Analyses of the National Institute on Aging Late-Onset Alzheimer's Disease Family Study: implication of additional loci. *Arch Neurol*. 2008;65(11):1518-1526. doi:10.1001/archneur.65.11.1518

28. Auria L, Moro RA. Support Vector Machines (SVM) as a Technique for Solvency Analysis. Published online February 2008.

29. McNeish DM. Using Lasso for Predictor Selection and to Assuage Overfitting: A Method Long Overlooked in Behavioral Sciences. *Multivar Behav Res*. 2015;50(5):471-484. doi:10.1080/00273171.2015.1036965

30. Chen X, Ishwaran H. Random forests for genomic data analysis. *Genomics*. 2012;99(6):323-329. doi:10.1016/j.ygeno.2012.04.003

31. Gross AL, Mungas DM, Leoutsakos J-MS, Albert MS, Jones RN. Alzheimer's disease severity, objectively determined and measured. *Alzheimers Dement Amst Neth*. 2016;4:159-168. doi:10.1016/j.dadm.2016.08.005

32. Karanicolas PJ, Farrokhyar F, Bhandari M. Practical tips for surgical research: blinding: who, what, when, why, how? *Can J Surg J Can Chir*. 2010;53(5):345-348.

33. Davis-Turak J, Courtney SM, Hazard ES, et al. Genomics pipelines and data integration: challenges and opportunities in the research setting. *Expert Rev Mol Diagn*. 2017;17(3):225-237. doi:10.1080/14737159.2017.1282822

34. Varma AR, Snowden JS, Lloyd JJ, Talbot PR, Mann DM, Neary D. Evaluation of the NINCDS-ADRDA criteria in the differentiation of Alzheimer's disease and

For Review Only

frontotemporal dementia. *J Neurol Neurosurg Psychiatry*. 1999;66(2):184-188. doi:10.1136/jnnp.66.2.184

35. McKhann GM, Knopman DS, Chertkow H, et al. The diagnosis of dementia due to Alzheimer's disease: recommendations from the National Institute on Aging-Alzheimer's Association workgroups on diagnostic guidelines for Alzheimer's disease. *Alzheimers Dement J Alzheimers Assoc*. 2011;7(3):263-269. doi:10.1016/j.jalz.2011.03.005

36. van der Ploeg T, Austin PC, Steyerberg EW. Modern modelling techniques are data hungry: a simulation study for predicting dichotomous endpoints. *BMC Med Res Methodol*. 2014;14:137. doi:10.1186/1471-2288-14-137

37. Chang Y, Wu J, Hong M-Y, et al. GenEpi: gene-based epistasis discovery using machine learning. Published online February 2020.

38. Powell M, Hosseini M, Collins J, et al. *I TRIED A BUNCH OF THINGS: THE DANGERS OF UNEXPECTED OVERFITTING IN CLASSIFICATION*. Neuroscience; 2016. doi:10.1101/078816

39. Vabalas A, Gowen E, Poliakoff E, Casson AJ. Machine learning algorithm validation with a limited sample size. *PloS One*. 2019;14(11):e0224365. doi:10.1371/journal.pone.0224365

40. Varma S, Simon R. Bias in error estimation when using cross-validation for model selection. *BMC Bioinformatics*. 2006;7:91. doi:10.1186/1471-2105-7-91

41. Zhou T, Liu M, Thung K-H, Shen D. Latent Representation Learning for Alzheimer's Disease Diagnosis With Incomplete Multi-Modality Neuroimaging and Genetic Data. *IEEE Trans Med Imaging*. 2019;38(10):2411-2422. doi:10.1109/TMI.2019.2913158

42. An L, Adeli E, Liu M, Zhang J, Lee S-W, Shen D. A Hierarchical Feature and Sample Selection Framework and Its Application for Alzheimer's Disease Diagnosis. *Sci Rep*. 2017;7(1):45269. doi:10.1038/srep45269

43. Ramspek CL, Jager KJ, Dekker FW, Zoccali C, van Diepen M. External validation of prognostic models: what, why, how, when and where? *Clin Kidney J*. 2021;14(1):49-58. doi:10.1093/ckj/sfaa188

44. Vardarajan BN, Faber KM, Bird TD, et al. Age-Specific Incidence Rates for Dementia and Alzheimer Disease in NIA-LOAD/NCRAD and EFIGA Families: National Institute on Aging Genetics Initiative for Late-Onset Alzheimer Disease/National Cell Repository for Alzheimer Disease (NIA-LOAD/NCRAD) and Estudio Familiar de Influencia Genetica en Alzheimer (EFIGA). *JAMA Neurol*. 2014;71(3):315. doi:10.1001/jamaneurol.2013.5570

45. El Hamid M, Omar Y, Mabrouk M. Identifying genetic biomarkers associated to Alzheimer's disease using Support Vector Machine. *IEEE*. Published online December 2016.

46. Abd El Hamid MM, Mabrouk MS, Omar YMK. DEVELOPING AN EARLY PREDICTIVE SYSTEM FOR IDENTIFYING GENETIC BIOMARKERS

For Review Only

ASSOCIATED TO ALZHEIMER'S DISEASE USING MACHINE LEARNING TECHNIQUES. *Biomed Eng Appl Basis Commun*. 2019;31(05):1950040. doi:10.4015/S1016237219500406

47. Namipashaki A, Razaghi-Moghadam Z, Ansari-Pour N. The Essentiality of Reporting Hardy-Weinberg Equilibrium Calculations in Population-Based Genetic Association Studies. *Cell J*. 2015;17(2):187-192. doi:10.22074/cellj.2016.3711

48. Sherif FF, Zayed N, Fakhr M. Discovering Alzheimer Genetic Biomarkers Using Bayesian Networks. *Adv Bioinforma*. 2015;2015:1-8. doi:10.1155/2015/639367

49. Samala RK, Chan H, Hadjiiski L, Helvie MA. Risks of Feature Leakage and Sample Size Dependencies in Deep Feature Extraction for Breast Mass Classification. *Med Phys*. Published online December 23, 2020:mp.14678. doi:10.1002/mp.14678

50. Probst P, Bischl B, Boulesteix A-L. Tunability: Importance of Hyperparameters of Machine Learning Algorithms. *ArXiv180209596 Stat*. Published online October 22, 2018. Accessed January 14, 2021. http://arxiv.org/abs/1802.09596

51. Bi X, Cai R, Wang Y, Liu Y. Effective Diagnosis of Alzheimer's Disease via Multimodal Fusion Analysis Framework. *Front Genet*. 2019;10:976. doi:10.3389/fgene.2019.00976

52. De Velasco Oriol J, Vallejo EE, Estrada K, Taméz Peña JG, Disease Neuroimaging Initiative TA. Benchmarking machine learning models for late-onset alzheimer's disease prediction from genomic data. *BMC Bioinformatics*. 2019;20(1):709. doi:10.1186/s12859-019-3158-x

53. Baker E, Escott-Price V. Polygenic Risk Scores in Alzheimer's Disease: Current Applications and Future Directions. *Front Digit Health*. 2020;2:14. doi:10.3389/fdgth.2020.00014

54. Ali A, Shamsuddin S, L.Ralesca A. Classification with class imbalance problem: a review. Published online November 2013.

55. Sun S, Cao Z, Zhu H, Zhao J. A Survey of Optimization Methods from a Machine Learning Perspective. *ArXiv190606821 Cs Math Stat*. Published online October 23, 2019. Accessed January 14, 2021. http://arxiv.org/abs/1906.06821

56. Cervantes J, Garcia-Lamont F, Rodriquez-Mazahua L, Lopez A. A comprehensive survey on support vector machine classification: Applications, challenges and trends. *Elsevier*. 2020;408:189-215.

57. Langley P, Sage S. Induction of Selective Bayesian Classifiers. *ArXiv13026828 Cs Stat*. Published online February 27, 2013. Accessed January 14, 2021. http://arxiv.org/abs/1302.6828

58. Misra S, Li H. Noninvasive fracture characterization based on the classification of sonic wave travel times. In: *Machine Learning for Subsurface Characterization*. Elsevier; 2020:243-287. doi:10.1016/B978-0-12-817736-5.00009-0

For Review Only

59. Wyner AJ, Olson M, Bleich J, Mease D. Explaining the Success of AdaBoost and Random Forests as Interpolating Classifiers. *ArXiv150407676 Cs Stat*. Published online April 29, 2017. Accessed January 14, 2021. http://arxiv.org/abs/1504.07676

60. Pretorius A, Bierman S, Steel S. A meta-analysis of research in random forests for classification. Published online November 2016.

61. Kumar ErP, Sharma ErP. Artificial Neural Networks-A Study. *Int J Emerg Eng Reseacrh Technol*. Published online May 2014.

62. Pavlou M, Ambler G, Seaman SR, et al. How to develop a more accurate risk prediction model when there are few events. *BMJ*. 2015;351:h3868. doi:10.1136/bmj.h3868

63. Srivastava N, Hinton G, Krizhevsky A, Salakhutdinov R. Dropout: A Simple Way to Prevent Neural Networks from Overfitting. *J Mach Learn Res*. Published online June 2014.

64. Weerts HJP, Mueller AC, Vanschoren J. Importance of Tuning Hyperparameters of Machine Learning Algorithms. *ArXiv200707588 Cs Stat*. Published online July 15, 2020. Accessed January 14, 2021. http://arxiv.org/abs/2007.07588

65. Ghojogh B, Crowley M. The Theory Behind Overfitting, Cross Validation, Regularization, Bagging, and Boosting: Tutorial. *ArXiv190512787 Cs Stat*. Published online May 28, 2019. Accessed January 14, 2021. http://arxiv.org/abs/1905.12787

66. Yadav S, Shukla S. Analysis of k-Fold Cross-Validation over Hold-Out Validation on Colossal Datasets for Quality Classification. Published online February 2016.

67. for the Alzheimer's Disease Neuroimaging Initiative, Hao X, Yao X, et al. Identifying Multimodal Intermediate Phenotypes Between Genetic Risk Factors and Disease Status in Alzheimer's Disease. *Neuroinformatics*. 2016;14(4):439-452. doi:10.1007/s12021-016-9307-8

68. Ibrahim AM, Bennett B. The Assessment of Machine Learning Model Performance for Predicting Alluvial Deposits Distribution. *Procedia Comput Sci*. 2014;36:637-642. doi:10.1016/j.procs.2014.09.067

69. Steyerberg EW, Vickers AJ, Cook NR, et al. Assessing the performance of prediction models: a framework for traditional and novel measures. *Epidemiol Camb Mass*. 2010;21(1):128-138. doi:10.1097/EDE.0b013e3181c30fb2

70. Park Y, Ho JC. Calibrated Random Forest for Health Data. Published online April 2020.

71. Riley RD, Snell KI, Ensor J, et al. Minimum sample size for developing a multivariable prediction model: PART II - binary and time-to-event outcomes. *Stat Med*. 2019;38(7):1276-1296. doi:10.1002/sim.7992

72. Blagus R, Lusa L. SMOTE for high-dimensional class-imbalanced data. *BMC Bioinformatics*. 2013;14(1):106. doi:10.1186/1471-2105-14-106

For Review Only

73. Morais R, Vasconcelos G. Under-Sampling the Minority Class to Improve the Performance of Over-Sampling Algorithms in Imbalanced Data Sets. Published online August 2017.

74. Parvandeh S, Yeh H-W, Paulus MP, McKinney BA. *Consensus Features Nested Cross-Validation*. Bioinformatics; 2020. doi:10.1101/2019.12.31.891895

75. Martin GP, Mamas MA, Peek N, Buchan I, Sperrin M. Clinical prediction in defined populations: a simulation study investigating when and how to aggregate existing models. *BMC Med Res Methodol*. 2017;17(1):1. doi:10.1186/s12874-016-0277-1

76. Haga SB. Impact of limited population diversity of genome-wide association studies. *Genet Med*. 2010;12(2):81-84. doi:10.1097/GIM.0b013e3181ca2bbf

77. Learned K, Durbin A, Currie R, et al. Barriers to accessing public cancer genomic data. *Sci Data*. 2019;6(1):98. doi:10.1038/s41597-019-0096-4

78. Cioffi R, Travaglioni M, Piscitelli G, Petrillo A, De Felice F. Artificial Intelligence and Machine Learning Applications in Smart Production: Progress, Trends, and Directions. *Sustainability*. 2020;12(2):492. doi:10.3390/su12020492

79. Wahed M, Sheriff F, Zayed N, Kadah Y. Integrated Higher-Order Evidence-Based Framework for Prediction of Higher-Order Epistasis Interactions in Alzheimer's Disease. Published online January 2017.

80. Zhang Z, Huang H, Shen D, Alzheimer's Disease Neuroimaging Initiative. Integrative analysis of multi-dimensional imaging genomics data for Alzheimer's disease prediction. *Front Aging Neurosci*. 2014;6. doi:10.3389/fnagi.2014.00260

For Review Only

Abbreviated Summary

This review aimed to assess the literature regarding the performance of machine learning algorithms for the prediction of Alzheimer's disease using genetic data. Publications were screened systematically for inclusion, with 12 studies passing all criteria set. Common issues such as the overreliance on one data source, insufficient sample sizes and inconsistent model performance reporting methods were outlined.

For Review Only
