# Supplementary material for: Machine learning for the life-time risk prediction of Alzheimer’s disease: a systematic review
Source: Brain Commun. 2021 Oct 21;3(4):fcab246. doi: 10.1093/braincomms/fcab246 (PMC8598986; doi:10.1093/braincomms/fcab246)
Supplement: fcab246_Supplementary_Data [file fcab246_supplementary_data.zip › supplementary_tables.docx]

Supplementary Table 1. Summary of the reviewed publications.

| Publication title and authors/publication date^a^ | Machine learning approaches^b^ | AUC for models^c^ | Accuracy for models^d^ | Data source used^e^ | Sample size^f^ |
| --- | --- | --- | --- | --- | --- |
| 1.  Benchmarking machine learning models for late-onset Alzheimer's disease prediction from genomic data  ^52^ | 2LASSO, 2RF, 2RPART, 2KNN, 2SVM (no-kernel),2 ensemble of all methods, 2BSWIMS = Linear models | (0.494-0.719) | N/A | ADNI SNPs only | Discover dataset:  230 Cases  241 Controls  Validation dataset:  37 Cases  130 Controls |
| 2.  Effective Diagnosis of Alzheimer’s Disease via Multimodal Fusion Analysis Framework  ^51^ | 5 SVMS kernel unspecified | N/A | Accuracy (0.70-0.87) | ADNI MRI and SNPs | 37 Cases  35 Controls |
| 3.  Latent Representation Learning for Alzheimer's Disease Diagnosis with Incomplete Multi-Modality Neuroimaging and Genetic Data  ^41^ | 9 SVMs kernel unspecified | (0.62-0.65) | Accuracy (0.59 – 0.67) | ADNI 1 MR images and SNPs | 171 cases  204 Controls |
| 4.  Discovering Alzheimer Genetic Biomarkers Using Bayesian Networks  ^48^ | NB, TAN NB, Markov blanket, minimal augmented markov blanket | N/A | Accuracy (0.62-0.66) | ADNI – SNPs only | 282 Controls  48 Cases |
| 5.  The application of naive Bayes model averaging to predict Alzheimer's disease from genome-wide data  ^24^ | NB, FSNB, MANB | (0.59-0.72) | N/A | GWAS collected and analysed originally by Reiman (M Reiman et al, 2008)  LOAD  GWAS – SNPs only | 550 Controls  861 Cases |
| 6.  A Hierarchical Feature and Sample Selection Framework and Its Application for Alzheimer's Disease Diagnosis  ^42^ | 5 SVMS all linear | (85.5-0.97)  SNPs only model = 85.5  MR + SNP model = 97.4 |  | ADNI 1 – MRI and SNPs | 204 Controls  171 Cases |
| 7  Integrated higher-order evidence-based framework for prediction of higher-order epistasis interactions in Alzheimer's disease  ^79^ | NB, RF, KNN, LR, SVM (rbf), multi-factor dimensionality reduction | N/A | Accuracy (0.62-0.78) | ADNI – SNPs only | 306 Cases  125 Controls |
| 8.  Integrative analysis of multi-dimensional imaging genomics data for Alzheimer’s disease prediction  ^80^ | 4 SVMS - all linear | N/A | Accuracy (0.88-0.95) | ADNI MRI and SNPs | 49 Cases  47 Controls |
| 9.  Identifying genetic biomarkers associated to Alzheimer's disease using Support Vector Machine  ^45^ | 10 SVMS 2 linear, 2 quadratic polynomial, 2 cubic polynomial, 2 RBF, 2PUK | N/A | Accuracy (0.62-0.77) | ADNI 1 SNPs only | 214 Controls  177 Cases |
| 10.  Improving predictive models for Alzheimer's disease using GWAS data by incorporating misclassified samples modelling  ^26^ | 2BSWiMS-logistic, 2GALGO-SVM (no-kernel). 2LASSO  8 LASSOs | (0.68-0.844) | N/A | National Institute on Aging—Late-Onset Alzheimer’s Disease  SNPs only | 2000 Controls  1856 Cases |
| 11.  GenEpi: Gene-based Epistasis Discovery Using Machine Learning  ^37^ | 3 Lasso regressions | N/A | Accuracy (0.83-0.94) | ADNI SNPs only | 241 Controls  123 Cases |
| 12.  Developing an early predictive system for identifying genetic biomarkers associated to Alzheimer’s disease using machine learning techniques  ^46^ | 2 SVM(Linear-kernel), 2 SVM(Quadratic-polynomial), 2 SVM(Cubic-polynomial), 2 Naïve Bayes, 2 Naïve Bayes(tree-augmented) and 2 Bayesian networks (K2) | N/A | Accuracy (0.95-0.99) | ADNI 1 data set. Also, a separate ADNI whole genome sequencing data set, genotyped using Illumina Omni 2.5 M.  SNPs only | 214 Controls  177 Cases  321 Controls  49 Cases |

Supplementary Table 2. Sensitivity and specificity values per publication.

| Publication title | Sensitivity | Specificity | Precision |
| --- | --- | --- | --- |
| Benchmarking machine learning models for late-onset Alzheimer’s disease prediction from genomic data | 0.033 – 0.719 | 0.62 – 0.981 | N/A |
| Effective Diagnosis of Alzheimer’s Disease via Multimodal Fusion Analysis Framework | N/A | N/A | N/A |
| Latent Representation Learning for Alzheimer’s Disease Diagnosis with Incomplete Multi-Modality Neuroimaging and Genetic Data | N/A | N/A | N/A |
| Discovering Alzheimer Genetic Biomarkers Using Bayesian Networks | 0.59-0.89 | 0.16 – 0.66 | N/A |
| The application of naïve Bayes model averaging to predict Alzheimer’s disease from genome-wide data | N/A | N/A | N/A |
| A Hierarchical Feature and Sample Selection Framework and Its Application for Alzheimer’s Disease Diagnosis | 0.75 – 0.86 | 0.85 – 0.96 | N/A |
| Integrated higher-order evidence-based framework for prediction of higher-order epistasis interactions in Alzheimer’s disease | 0.62 – 0.75 | 0.55 – 0.82 | N/A |
| Integrative analysis of multi-dimensional imaging genomics data for Alzheimer’s disease prediction | 0.90 – 0.94 | 0.85 – 0.96 | N/A |
| Identifying genetic biomarkers associated to Alzheimer's disease using Support Vector Machine | 0.62 – 0.77 | N/A | 0.59 – 0.67 |
| Improving predictive models for Alzheimer’s disease using GWAS data by incorporating misclassified samples modelling | 0.61-0.83 | 0.73 – 0.86 | N/A |
| GenEpi: Gene-based Epistasis Discovery Using Machine Learning | 0.66 – 0.85 | N/A | 0.77 – 0.96 |
| Developing an early predictive system for identifying genetic biomarkers associated to Alzheimer’s disease using machine learning techniques | 0.57 – 0.98 | .  N/A | 0.59 – 1.00 |

Supplementary Table 3. Methods of Validation used.

| Methods^a^ | Number of Studies^b^ | Number of Models^c^ |
| --- | --- | --- |
| Cross-validation – Number of folds not specified. | 1 | 14 |
| Cross-validation – 20 Folds | 1 | 14 |
| Cross-validation – 10 Folds | 7 | 50 |
| Cross-validation – 5 Folds | 1 | 3 |
| Cross-validation – 2 Folds | 1 | 2 |
| Leave-one-out (LOO) CV | 1 | 1 |
| Training/Test split 60:40 | 1 | 5 |

^.^

Supplementary Table 4. Class imbalances for each study.

| Publication title^a^ | Number of Controls^b^ | Number of Cases^c^ | Class Imbalance^d^ |
| --- | --- | --- | --- |
| Benchmarking machine learning models for late-onset Alzheimer’s disease prediction from genomic data | Discovery dataset: 230  Validation dataset: 130 | Discovery dataset: 241  Validation dataset: 37 | 0.954  3.514 |
| Effective Diagnosis of Alzheimer’s Disease via Multimodal Fusion Analysis Framework | 35 | 37 | 0.946 |
| Latent Representation Learning for Alzheimer’s Disease Diagnosis with Incomplete Multi-Modality Neuroimaging and Genetic Data | 204 | 171 | 1.193 |
| Discovering Alzheimer Genetic Biomarkers Using Bayesian Networks | 282 | 48 | 5.875 |
| The application of naïve Bayes model averaging to predict Alzheimer’s disease from genome-wide data | 550 | 861 | 0.639 |
| A Hierarchical Feature and Sample Selection Framework and Its Application for Alzheimer’s Disease Diagnosis | 204 | 171 | 1.193 |
| Integrated higher-order evidence-based framework for prediction of higher-order epistasis interactions in Alzheimer’s disease | 125 | 306 | 0.408 |
| Integrative analysis of multi-dimensional imaging genomics data for Alzheimer’s disease prediction | 47 | 49 | 0.959 |
| Identifying genetic biomarkers associated to Alzheimer's disease using Support Vector Machine | 214 | 177 | 1.209 |
| Improving predictive models for Alzheimer’s disease using GWAS data by incorporating misclassified samples modelling | 2000 | 1856 | 1.078 |
| GenEpi: Gene-based Epistasis Discovery Using Machine Learning | 241 | 132 | 1.826 |
| Developing an early predictive system for identifying genetic biomarkers associated to Alzheimer’s disease using machine learning techniques | 214  321 | 177  49 | 1.209  6.551 |

Supplementary Table 5. Information extracted for the type of predictive materials used

and methods for the pre-processing of SNPs.

| Publication title^a^ | Types of data modality used^c^ | SNPs QC General | MAF | Missing value rate |
| --- | --- | --- | --- | --- |
| Benchmarking machine learning models for late-onset Alzheimer’s disease prediction from genomic data | SNPs | Marker call rate - ≤ 99%  Hardy Weinberg Equilibrium test - ≤ 0.05  LD based clumping – p-value ≤ 0.01 and r2 ≤ 0.05. | ≤ 0.01 | N/A |
| Effective Diagnosis of Alzheimer’s Disease via Multimodal Fusion Analysis Framework | SNPs/MRI | Sample call rate – 95%  Genotyping – 99.9%  Hardy Weinberg test 0.0001 % | 4% | N/A |
| Latent Representation Learning for Alzheimer’s Disease Diagnosis with Incomplete Multi-Modality Neuroimaging and Genetic Data | SNPs/MRI/PET  (Positron emitting tomography) | Selected SNPs were imputed to estimate missing genotypes. Illumina annotation information was used to select a subset of SNPs |  |  |
| Discovering Alzheimer Genetic Biomarkers Using Bayesian Networks | SNPs | Hardy Weinberg test ≤ 0.001 | 0.01 |  |
| The application of naïve Bayes model averaging to predict Alzheimer’s disease from genome-wide data | SNPs | N/A | N/A | N/A |
| A Hierarchical Feature and Sample Selection Framework and Its Application for Alzheimer’s Disease Diagnosis | SNPs/MRI | Gender Check  Hardy Weinberg Equilibrium test  Population Stratification | Percentage not specified |  |
| Integrated higher-order evidence-based framework for prediction of higher-order epistasis interactions in Alzheimer’s disease | SNPs | N/A | N/A | N/A |
| Integrative analysis of multi-dimensional imaging genomics data for Alzheimer’s disease prediction | SNPs/MRI/PET/CSF  (Cerebrospinal fluid) | Call rate check per subject, gender check,  The Hardy Weinberg Equilibrium test, Population stratification | Percentage not specified |  |
| Identifying genetic biomarkers associated to Alzheimer's disease using Support Vector Machine | SNPs | Removing individuals with discordant gender information, LD pruning, subjects with high IBD are removed, Hardy Weinberg Equilibrium test ≤ 0.000005 %. | ≤ 0.001 | ≤ 10% |
| Improving predictive models for Alzheimer’s disease using GWAS data by incorporating misclassified samples modelling | SNPs | Marker call rate removal ≤ 98%, monomorphic markers also removed, Hardy Weinberg Equilibrium rate ≤ 0.000. | N/A | N/A |
| GenEpi: Gene-based Epistasis Discovery Using Machine Learning | SNPs | Missing data imputation according to the 1000 genome haplotypes. |  |  |
| Developing an early predictive system for identifying genetic biomarkers associated to Alzheimer’s disease using machine learning techniques | .  SNPs | Removing individuals with discordant gender information, LD pruning, subjects with high IBD are removed, Hardy Weinberg Equilibrium test ≤ 0.000005 %. | ≤ 0.01 | ≤ 10% |

Supplementary Table 6. Methods to deal with missing data

| Publication title | Were methods used to deal with missing data. | Missing data Methods used. | Hyperparameter Tuning Methods^b^ |
| --- | --- | --- | --- |
| Benchmarking machine learning models for late-onset Alzheimer’s disease prediction from genomic data | No | N/A | N/A |
| Effective Diagnosis of Alzheimer’s Disease via Multimodal Fusion Analysis Framework | No | N/A | Model aspects such as number of trees in RF and tree inputs were optimised. |
| Latent Representation Learning for Alzheimer’s Disease Diagnosis with Incomplete Multi-Modality Neuroimaging and Genetic Data | Yes | The formulation of a latent representation learning method, which used incomplete samples. | Hyperparameters were tuned using CV |
| Discovering Alzheimer Genetic Biomarkers Using Bayesian Networks | Yes | Missing values imputed by Expectation Maximization algorithm. | N/A |
| The application of naïve Bayes model averaging to predict Alzheimer’s disease from genome-wide data | No | N/A | N/A |
| A Hierarchical Feature and Sample Selection Framework and Its Application for Alzheimer’s Disease Diagnosis | Yes | Missing genotypes were imputed, no method given. | Parameters for feature selection method were optimised using CV |
| Integrated higher-order evidence-based framework for prediction of higher-order epistasis interactions in Alzheimer’s disease | No | N/A | N/A |
| Integrative analysis of multi-dimensional imaging genomics data for Alzheimer’s disease prediction | Yes | Missing genotypes were imputed using MaCH software. | Parameters of SVM tuned using grid search |
| Identifying genetic biomarkers associated to Alzheimer's disease using Support Vector Machine | Yes | Those samples who had greater than 10% of samples missing were discarded. | N/A |
| Improving predictive models for Alzheimer's disease using GWAS data by incorporating misclassified samples modelling | Yes | Missing values replaced with median of nearest neighbours | CV was used for the tuning of lambda hyperparameter |
| GenEpi: Gene-based Epistasis Discovery Using Machine Learning | Yes | Missing genotypes imputed according to the 1000 genome haplotypes | N/A |
| Developing an early predictive system for identifying genetic biomarkers associated to Alzheimer’s disease using machine learning techniques | Yes | Those samples who had greater than 10% of samples missing were discarded. | N/A |

Supplementary Table 7. Descriptive statistics if reported.

| Publication title^a^ | Age^b^ | Gender^c^ |
| --- | --- | --- |
| Benchmarking machine learning models for late-onset Alzheimer's disease prediction from genomic data | Discovery dataset:  Mean age – 75.57  Validation dataset:  Mean age – 72.17 | Discovery:  Males – 252, Females – 219  1.15    Validation:  Males – 92, Females - 75  1.23 |
| Effective Diagnosis of Alzheimer’s Disease via Multimodal Fusion Analysis Framework | Cases:  Mean age - 75.35  Controls:  Mean age – 77.14 | Cases:  Males – 19, Females – 18  1.05  Controls:  Males – 13, Females – 22  0.59 |
| Latent Representation Learning for Alzheimer's Disease Diagnosis with Incomplete Multi-Modality Neuroimaging and Genetic Data | Cases:  Mean age – 75.5  Controls:  Mean age – 76.1 | Cases:  Males – 94, Females – 77  1.22  Controls:  Males – 112, Females – 92  1.33 |
| Discovering Alzheimer Genetic Biomarkers Using Bayesian Networks | N/A | N/A |
| The application of naive Bayes model averaging to predict Alzheimer's disease from genome-wide data | N/A | N/A |
| A Hierarchical Feature and Sample Selection Framework and Its Application for Alzheimer's Disease Diagnosis | Cases:  Mean age – 75.5  Controls:  Mean age – 76.1 | Cases:  Males – 94, Females – 77  1.22  Controls:  Males – 112, Females – 92  1.22 |
| Integrated higher-order evidence-based framework for prediction of higher-order epistasis interactions in Alzheimer’s disease | N/A | N/A |
| Integrative analysis of multi-dimensional imaging genomics data for Alzheimer’s disease prediction | N/A | N/A |
| Identifying genetic biomarkers associated to Alzheimer's disease using Support Vector Machine | N/A | N/A |
| Improving predictive models for Alzheimer's disease using GWAS data by incorporating misclassified samples modelling | N/A | N/A |
| GenEpi: Gene-based Epistasis Discovery Using Machine Learning | N/A | N/A |
| Developing an early predictive system for identifying genetic biomarkers associated to Alzheimer’s disease using machine learning techniques | N/A | N/A |

Legends:

Supplementary Table 1. Summary of the reviewed publications.

^a – Publication title; b – The range of sensitivity values detailed within each study. – c AUC values reported by models; - d ACC values reported for models; - e The source of data used in the study; - f the sample size used by cases and controls.^

Supplementary Table 2. Sensitivity and specificity values per publication.

^a – Publication title; b – The range of sensitivity values detailed within each study.^

^c – The range of specificity values detailed within each study. D – Values for precision if reported.^

Supplementary Table 3. Methods of Validation used.

^a – Publication title; b – The range of sensitivity values detailed within each study.^

^c – The range of specificity values detailed within each study^

Supplementary Table 4. Class imbalances for each study.

^a – Publication title; b – The number of controls in the study; c – The number of cases in the study; d – The ratio of cases versus controls – Imbalance.^

Supplementary Table 5. Information extracted for the type of predictive materials used

and methods for the pre-processing of SNPs.

^a – Publication title; b – Hyperparameter tuning methods.^

^c – Types of data modality used; d – Pre-processing steps for SNPs.^

Supplementary Table 6. Methods to deal with missing data

^a – Publication title; b – Whether methods were employed to deal with missing data.^

^c – Methods used to deal with missing data.^

Supplementary Table 7. Descriptive statistics.

^a – Publication title; b – The mean age of study participants, recorded either by case/control split or by different datasets.^

^c – The breakdown of males/females for participants, by case/control split or by dataset. With the ratio of males to females.^
