# Supplementary material for: Machine learning for the life-time risk prediction of Alzheimer’s disease: a systematic review
Source: Brain Commun. 2021 Oct 21;3(4):fcab246. doi: 10.1093/braincomms/fcab246 (PMC8598986; doi:10.1093/braincomms/fcab246)
Supplement: fcab246_Supplementary_Data [file Author Rebuttal.pdf › Author Rebuttal.pdf]

Comments:
1. The authors have mentioned one similar review in this field in the introduction part. Can the authors check any other review papers in this field? Although some may have a different focus, such as using neuroimaging data instead of genetic data. Compared with previous reviews, what is the primary focus and contribution of this review?
• Tanveer, M., et al. “Machine learning techniques for the diagnosis of Alzheimer’s disease: A review.” ACM Transactions on Multimedia Computing, Communications, and Applications (TOMM) 16.1s (2020): 1-35.
• Khan, Aunsia, and Muhammad Usman. “Early diagnosis of Alzheimer’s disease using machine learning techniques: A review paper.” 2015 7th International Joint Conference on Knowledge Discovery, Knowledge Engineering and Knowledge Management (IC3K). Vol. 1. IEEE, 2015.

A summary of other reviews and a comparison with this review would certainly contribute to this research. We conducted a search for other similar reviews in this field. Therefore, we conducted a search for other similar reviews in this field. Those found focused on imaging data only. However, the results were similar to those found in this review. The most notable difference was the absence of any calculation of ROB in the other reviews. Therefore, this research has gone into greater detail in terms of model implementation, data used and performance reporting. This gives it a unique perspective in the current field. A paragraph was added to the original text (pages 26, lines 715-722) to highlight previous reviews.

As well as the above input, an extra sentence was also added to the introduction (pages 4-5, lines 130-33). This reiterated that whilst other reviews have been conducted in this area, this review is unique in its assessment of bias.

2. In the title, “risk prediction” was used as the endpoint of the studies under review. However, it would be helpful if the problem setting can be explained in the abstract or introduction. We assume it is not referring to diagnose AD at the current time point using the cross-sectional data. “Risk prediction” can be vague, such as the prognosis prediction using longitudinal data or whether the patient will develop AD at a later time point using the current cross-sectional data. It seems the authors focused on the latter scenario in this review. A clear definition of the input (cross-sectional data, or longitudinal data) and output (AD diagnosis at the current time point, the risk of getting AD at a later time point, or the risk of deterioration) would be preferred.

We agree that the term ‘risk prediction’ has a broad definition. The original literature search used a number of terms within a defined window of time. The resulting papers were then screened to determine whether they matched our pre-defined acceptance criteria. This criterion did not specify whether authors should have used either cross-sectional or longitudinal data, therefore both would have been included. Unfortunately, there were no instances of longitudinal data usage in the accepted for review papers. To clarify this position further, we have added clarification in the second paragraph of the Introduction (page 3, lines 83-84) that included a definition the term risk prediction. We also specified that the review assessed studies which used cross-sectional data to determine an individual’s risk of developing AD at any point.

3. Why focus on SNP features only? Is there any genomic data provided by the ADNI dataset? It would also be recommended to reflect the focus of SNP features in the title.

SNPs were the only form of genetic variation accepted to facilitate comparisons between the studies, therefore articles focusing on gene expression data or other forms of genetic data (e.g., rare variants) were not included. We have added clarification on pages 5-6, lines 157-159 and 163-165.

4. How to understand the calibration of the models (Page 6-7)?

We agree that model calibration required greater explanation. We have added further clarification where the term was first introduced (page 7, lines 214-217). Previous systematic reviews have shown that calibration has

historically been scarcely reported. Further to this, we have also included a brief discussion on why poorly calibrated models can cause issues in the healthcare sector.

5. Only 12 studies were included for the review. Can the authors discuss why only a few studies have been performed in this study? Is this because the field is not ready?

We agree that 12 studies could be perceived as a small number of studies, although this is in line with other ML reviews regarding healthcare prediction (page 21, lines 524-529) (e.g., only 13 studies were included in the ML in a Schizophrenia review paper with a similar search strategy (see Bracher-Smith *et al*. (2021). Machine learning for genetic prediction of psychiatric disorders: a systematic review. Mol Psychiatry. 26(1):70-79)). We have updated the text to include several potential reasons why this is the case. These are the relatively recent emergence of ML approaches and the difficulty in securing permission for sufficient datasets.

6. For the inclusion criteria of this review, only binary prediction was included by the authors. It will be helpful if a justification could be provided to explain why selecting this endpoint. Is this because the SNPs cannot differentiate MCWE or AD?

We have added further clarification on why this was the case (page 6, lines 168-172). We included that a clear boundary between MCI and dementia patients does not exist, with clinicians often having to partly use opinion-based decisions to determine which category an individual resides in. Therefore, a decision was taken to remove this uncertainty and only use only cases and controls.

7. For section “3.2 Risk of Bias”, the authors mentioned that “ROB deemed low for participants,” as most publications used the ADNI dataset and assessed it in a cross-sectional format. However, the ADNI data contains several cohorts (ADNI 1, ADNI 2, and ADNI GO) and various data modalities (e.g., MR imaging, SNPs, and electronic health records). Did the papers under review use the same subset of ADNI data for their ML models? If not, will the different subsets of samples/features cause potential ROB?

We further examined the 10 papers which used ADNI. We concluded that four studies used ADNI 1 and the remaining texts did not specify the type of ADNI used. This information was included in the second paragraph of the results section (page 9, lines 263-264). Due to four studies using ADNI1 and the remaining not specifying, we concluded that this did not cause any ROB.

8. In the ROB for predictors, the authors mentioned, “However, time intervals between measurements differed for Alzheimer’s disease patients and those deemed either controls or MCI study participants … Therefore, this time interval difference was considered to be of low ROB.” Did the models use MCI patients for training and testing in the 12 studies under review? The authors also mentioned time intervals in writing. Did the model use any longitudinal data in these 12 studies under review? It would be helpful if the authors can clearly compare the inputs (number and type of features, number of patients, time points/intervals, etc.) for each model.

We agree that including information about MCI study participants is confusing as we have specified that only models using cases and controls were reviewed. Discussing collection procedures for MCI individuals was therefore not relevant. Also, models did not use any longitudinal data in the 12 papers included. Given these points, we removed this information from the review. Therefore, content was removed from the 2nd paragraph of the ROB section, this included discussion about the difference between collections for MCI and AD individuals. We also removed the text regarding the time intervals as no longitudinal data were used by any of the included studies. In addition, in the 2nd paragraph of the results section, we reiterated that no longitudinal data were used by any of the reviewed studies (page 9, lines 260-263).

9. The authors use Events per Variable (EPV) to evaluate the sample sizes used in the models. Can the authors use some visualization to compare the EPV of all studies under review?

We agree that a visualisation of EPV values would enhance the paper. A forest plot has now been included in the sample size section. This displays number of samples, number of predictors, AUC and ACC for papers and EPV values. Two scales have been used due to the large variance between some values.

10. In Fig. 2, it seems the number of samples and the number of features used in each study vary significantly from each other. Can the authors justify how to make a fair comparison of the model performance given such various sub-datasets (i.e., various samples and features)? In section "3.4 Sample Size", some studies use a different number of samples (e.g., "300-900 individuals from the ADNI data set"). Can the authors use a table or some writing to summarize the sample selection processes of these papers?

We agree that for clarity a justification is needed. Therefore, we have added more information at the end of the first paragraph in section 3.3 (page 14, lines 383-386). We added clarification on how confidence intervals in Figure 2 allow for comparison between the studies. This is due to the confidence intervals reflecting the variability of AUC with respect to sample size. Such calculation was not made for studies which reported ACC for their performance metric. Further comment has also been added in the discussion (page 24, lines 642-644), this gives a brief description of what was found regarding sample sizes and the number of features.

The selection of exact numbers of individuals for each study depends on the variables used and the version of ADNI. Studies did not provide reasons on why they selected certain numbers of samples; therefore, it was not possible to summarise it further.

12. In section "3.5 Predictors", the authors described the pre-processing techniques for SNPs. It would be helpful to provide a flow chart for the complete pre-processing pipelines for SNPs among these studies.

We agree that additional information was needed on SNP pre-processing methods in each study. Supplementary Table 5 has been updated and provides a more in-depth description of the pre-processing pipelines used by each article. This includes methods to account for relatedness between individuals, LD pruning and clumping.

13. Are there any machine learning techniques such as Minimum Redundancy Maximum Relevance (mRMR) applied for feature selection in these studies?

This is both an interesting and common feature selection technique used in genetic studies. However, only one of the 12 studies reviewed mentioned its use. We have added a short section to the discussion (pages 24 - 25, lines 654-659) that includes a given brief description on how the algorithm works and which study used it.

14. For the "hyperparameter search" section, can the authors use a table to show the hyper-parameters that need to be tuned for various machine learning models?

A summary of the various hyperparameters needed to be tuned would be a good addition to the paper. Therefore, we added an extra column to Supplementary Table 6 in which we detailed any hyperparameters used in the ML models.

15. In the "hyperparameter search" section, the authors mentioned that only one study applied nested cross-validation for hyperparameter search. For the other studies using a CV instead of a nested CV, how the hyperparameter search is achieved without an inner loop of CV?

We agree that this requires further clarification. The statement that only one study used nested CV was incorrect. We subsequently found that one further study also used this method. Therefore, the first paragraph of the hyperparameter tuning section has now been updated with details of this paper (page 20, lines 495-500). Also, the remaining 3 studies which did use hyperparameter tuning, did not specify whether nested CV was used or not. Therefore, it is difficult to speculate on how hyperparameters were tuned. This could be in the same CV loop as model optimisation, which would introduce ROB. However, Nested CV may have been used but simply not reported.

16. In the discussion section, the authors have discussed the pros and cons of various ML models (e.g., SVM, NB, and RFs). However, no clear conclusion has been drawn from this comparison. Can the authors provide a guideline of ML model selection for AD prediction using SNPs features? Various scenarios such as the number of SNP features, the number of samples, the presence of data issues such as missing values can be considered for the guideline. For example,

• Scenario A: 300 samples, 500 SNPs, which ML model should be used.
• Scenario B: 300 samples, 50,000 SNPs, which ML model should be used.

This is an interesting and important question for the field moving forward, however it is unfortunately out of the scope of this review. A recommendation such as this would be drawn from speculation and not information gathered from this review. Additional evidence such as further publications and simulation studies would be needed to answer this question.

17. Although the neural network is not compared in this review, some NN-based AD risk prediction using SNPs data has been published recently. They can be added to the discussion section if they are not in the original date range for the paper search.

After conducting an up-to-date search, we found two papers which used NNs to predict Alzheimer's disease using genetic data. The first of these did fall in our time window, but it was a preprint, and these were excluded from our review. However, despite this we have included it in the discussion, this follows the paragraph commenting on the lack of NNs in the review (page 22, lines 572-581). We explained the focus of the paper, the methods used, the results obtained and provided a summary of the conclusions drawn from the paper.

The second paper found was a peer-reviewed publication and again fell into our window of search. One possible reason for not including this paper could be the choice of keywords used. The terms neural network or deep learning were not part of our criteria. Therefore, this paper might not have been returned by our searches. This has now been listed as a limitation of the review. A brief description of this 2nd paper and its results have been given, as well as a discussion regarding potential biases in novel techniques. This information was added to page 22-23, lines 583-592.

18. The curse of dimensionality is a major challenge in omics study (e.g., SNPs). Can the authors briefly discuss the challenges and opportunities from the ML perspective for AD prediction in the discussion section?

The curse of dimensionality is a major challenge for ML prediction. This had been explained when discussing low EPV values and small sample size, however we did not use the official term. This has now been addressed in the discussion (page 24, lines 647-649). We introduced the term 'curse of dimensionality' and suggested how this is linked to low EPV values and sample sizes. We have also commented on the effect this could have on the modelling.

19. Although most studies use the ADNI dataset, it has some limitations, such as the small sample size (e.g., a few hundred) and population bias (e.g., most participants are Caucasian). As the quality of training data is

essential for any ML models' success, can the authors provide insights on any alternative to the ADNI dataset? Such as alternative AD datasets or computational methods for data augmentation.

We are not aware of any other easily accessible dataset. Due to data protection policies of sensitive data (and genetic data is categorised as such), most of the large genetic datasets require strict permissions and exact description of their use. We have further emphasised this point in the discussion (page 27, lines 742-749).

20. Based on the current discussion, can the authors summarize the challenges and opportunities of ML for AD risk prediction using genetic data? It would be helpful if the authors can provide some insights on the research priorities in this field (e.g., the collection of more data, the development of feature selection techniques, the improvements of classification techniques, or the development of model optimization techniques).

A summary of the challenges facing this research area is a good idea. We briefly commented on these challenges when discussing issues of sample size and the curse of dimensionality. We suggested that a wider range of data sources needed to be used, however barriers to gaining access to these do exist. Further information has now been added to the final paragraph of the paper (page 27, lines 742-749). This includes the need for improvement in classification and optimisation methods, as well as feature selection techniques. However, emphasis was placed on the need for larger datasets to achieve this.

Minor suggestions:
1. Numbering of the subsection "3.5 Hyperparameter search" should be 3.6.
2. Numbering of the subsection "3.6 Descriptive statistics" should be 3.7.
3. Grammar: comma before "and" in a list of objects.
For example, "the type of models used, model development, and the validity of the reported results."

Reviewer 2

1.The introduction mentions that the review will assess the ability of ML to predict AD using only genetic data, but in the next chapter the inclusion of multimodal data is included. This should be consistent. The abstract should also be modified to include the multimodal data. Furthermore, the Inclusion Criteria should also reflect this for clarity.

We agree that this was not consistent through both the abstract, introduction, and inclusion criteria. Therefore, changes have been made in order to make this clearer. In the abstract (page 1, lines 16-17), we clarified that studies which used SNP data only and those which combined SNPs with other types of data were both accepted. In the introduction, we have specified that SNP data were the primary data source used (page 4, lines 157-159). However, studies which combined SNP data with other forms of data were also accepted.

2. Further detail could be included into why an AUC of 0.8 using genetic data is an impressive metric due to the multifactorial complexity of the disease and the limits of the genetic component (Expanding into the value of multimodal analysis).

Additional information in this area would add value to this review article, therefore we have added information to where this specific paper was mentioned (page 21, lines 532-538). The impressiveness of the result was emphasised further by introducing that current prediction models used in other areas match this result. Also, comments were made on how difficult prediction of Alzheimer's disease can be considering its polygenic nature.

3. Including more insight into the missing heritability and the benefits of ML methods to solve multifactorial diseases should be considered.

We agree that further comment on missing heritability would enhance the paper. In the second paragraph of the introduction, we have added additional insights. We added that GWAS have not accounted for the heritability suggested by twin studies and that this amounts to a level of missing heritability (page 3, lines 77-84).

4. In tables and comparisons (specially for AUC and ACC), the comments section could be improved by making it clearer which studies use multimodal data and which are SNP-only. Additionally, a lack of SNP-preprocessing or a large class-imbalance could also be included.

We agree that further information is required on those studies which reported AUC /ACC and which of these used SNP only and multimodal data. We have included extra information to the 'Data Used' column of Supplementary Table 1. We provided information on whether the study used SNPs only or multimodal data. This gives a better of understanding on the relationship between ACC/AUC and the type of data used. Further information has now been added in the discussion. This gives a description of the differences in terms of AUC/ACC for studies which used SNPs only and those which used a multimodal approach (page 25, lines 669-673).
