# Supplementary material for: Machine learning for the life-time risk prediction of Alzheimer’s disease: a systematic review
Source: Brain Commun. 2021 Oct 21;3(4):fcab246. doi: 10.1093/braincomms/fcab246 (PMC8598986; doi:10.1093/braincomms/fcab246)
Supplement: fcab246_Supplementary_Data [file Decision Letter.pdf › Decision Letter.pdf]

Decision Letter

14-Apr-2021

Dear Dr. Escott-Price,

Thank you for your recent submission BRAINCOM-2021-086 entitled "Machine Learning for the Risk Prediction of Alzheimer's Disease: A Systematic Review" to Brain Communications.

Your manuscript has been reviewed, and while the reviewers and editorial team appreciate many aspects of the paper, they raise some constructive critiques that I hope you can address in a revision of the paper.

I invite you to submit a revised manuscript and a response to each of the reviewers' comments. The reviewer(s)' comments are included at the bottom of this message.

In addition to the reviewers' comments, please note our editorial comments added below to comply with our requirements for presentation of scientific data at Brain Communications.

Please format your revised manuscript according to our Instructions to Authors https://academic.oup.com/braincomms/pages/General_Instructions – a summary of key points is attached to help with this.

To revise your manuscript, click the link below or log into https://mc.manuscriptcentral.com/braincom and enter your Author Centre, where you will find your manuscript title listed under "Manuscripts with Decisions." Under "Actions," click on "Create a Revision." Your manuscript number will be appended to denote a revision.

*** PLEASE NOTE: This is a two-step process. After clicking on the link, you will be directed to a webpage to confirm. ***

https://mc.manuscriptcentral.com/braincom?URL_MASK=70ca87682fe24b399fb2e1dfc513f46e

Please upload a revised manuscript with changes tracked with track changes or in coloured text.

When submitting your revised manuscript, you will be asked to respond to the reviewer(s) comments. Please be as specific as possible in your response to all of the comments.

The editorial team at Brain Communications aims to increase transparency in scientific publishing and to demonstrate the value that peer review brings to the scientific process. Please therefore consider whether you are willing to have reviewer(s)' comments on your paper and your response published as supplementary data with the paper if it is accepted. This is entirely optional and will only be done if both the authors and reviewers agree. While submitting your revision there will be an opportunity to review or update the answer you gave previously, if necessary.

IMPORTANT: Your original files are available to you when you upload your revised manuscript. Please delete any redundant files before completing the submission.

Because we are trying to facilitate timely publication of manuscripts submitted to Brain Communications, we would appreciate your revised manuscript as soon as possible. If it is not possible for you to resubmit within 3 months, the option to submit a revision will expire, and we would then consider your revised paper as a new submission. However, if you need a little more time, please get in touch and we can extend this deadline for you.

Once again, thank you for submitting your manuscript to Brain Communications and I look forward to receiving your revision.

Yours sincerely,

Prof. Tara Spires-Jones
Editor, Brain Communications
Tara.Spires-Jones@ed.ac.uk

Reviewer(s)' Comments to Author:

Reviewer 1: Anonymous

Comments to the Author
In this paper, the authors have applied the PRISMA to systematically reviewed the machine learning (ML) models for the risk prediction of Alzheimer's disease (AD) using genetic data. The authors have scanned thousands of papers and identified 12 studies for comparison. The types of ML models, the data and data pre-processing, the training processes, and the model performances of these 12 studies have been carefully examined. Overall, the paper is well-written and easy to follow.

Comments:
1. The authors have mentioned one similar review in this field in the introduction part. Can the authors check any other review papers in this field? Although some may have a different focus, such as using neuroimaging data instead of genetic data. Compared with previous reviews, what is the primary focus and contribution of this review?
• Tanveer, M., et al. "Machine learning techniques for the diagnosis of Alzheimer's disease: A review." ACM Transactions on Multimedia Computing, Communications, and Applications (TOMM) 16.1s (2020): 1-35.
• Khan, Aunsia, and Muhammad Usman. "Early diagnosis of Alzheimer's disease using machine learning techniques: A review paper." 2015 7th International Joint Conference on Knowledge Discovery, Knowledge Engineering and Knowledge Management (IC3K). Vol. 1. IEEE, 2015.
2. In the title, "risk prediction" was used as the endpoint of the studies under review. However, it would be helpful if the problem setting can be explained in the abstract or introduction. I assume it is not referring to diagnose AD at the current time point using the cross-sectional data. "Risk prediction" can be vague, such as the prognosis prediction using longitudinal data or whether the patient will develop AD at a later time point using the current cross-sectional data. It seems the authors focused on the latter scenario in this review. A clear definition of the input (cross-sectional data, or longitudinal data) and output (AD diagnosis at the current time point, the risk of getting AD at a later time point, or the risk of deterioration) would be preferred.
3. Why focus on SNP features only? Is there any genomic data provided by the ADNI dataset? It would also be recommended to reflect the focus of SNP features in the title.
4. How to understand the calibration of the models (Page 6-7)?
5. Only 12 studies were included for the review. Can the authors discuss why only a few studies have been performed in this study? Is this because the field is not ready?
6. For the inclusion criteria of this review, only binary prediction was included by the authors. It will be helpful if a justification could be provided to explain why selecting this endpoint. Is this because the SNPs cannot differentiate MCI or AD?
7. For section "3.2 Risk of Bias", the authors mentioned that "ROB deemed low for participants," as most publications used the ADNI dataset and assessed it in a cross-sectional format. However, the ADNI data contains several cohorts (ADNI 1, ADNI 2, and ADNI GO) and various data modalities (e.g., MR imaging, SNPs, and electronic health records). Did the papers under review use the same subset of ADNI data for their ML models? If not, will the different subsets of samples/features cause potential ROB?
8. In the ROB for predictors, the authors mentioned, "However, time intervals between measurements differed for Alzheimer's disease patients and those deemed either controls or MCI study participants … Therefore, this time interval difference was considered to be of low ROB." Did the models use MCI patients for training and testing in the 12 studies under review? The authors also mentioned time intervals in writing. Did the model use any longitudinal data in these 12 studies under review? It would be helpful if the authors can clearly compare the inputs (number and type of features, number of patients, time points/intervals, etc.) for each model.
9. The authors use Events per Variable (EPV) to evaluate the sample sizes used in the models. Can the authors use some visualization to compare the EPV of all studies under review?
10. In Fig. 2, it seems the number of samples and the number of features used in each study vary significantly from each other. Can the authors justify how to make a fair comparison of the model performance given such various sub-datasets (i.e., various samples and features)?
11. In section "3.4 Sample Size", some studies use a different number of samples (e.g., "300-900 individuals from the ADNI data set"). Can the authors use a table or some writing to summarize the sample selection processes of these papers?
12. In section "3.5 Predictors", the authors described the pre-processing techniques for SNPs. It would be helpful to provide a flow chart for the complete pre-processing pipelines for SNPs among these studies.
13. Are there any machine learning techniques such as Minimum Redundancy Maximum Relevance (mRMR) applied for feature selection in these studies?
14. For the "hyperparameter search" section, can the authors use a table to show the hyper-parameters that need to be tuned for various machine learning models?
15. In the "hyperparameter search" section, the authors mentioned that only one study applied nested cross-validation for hyperparameter search. For the other studies using a CV instead of a nested CV, how the hyperparameter search is achieved without an inner loop of CV?
16. In the discussion section, the authors have discussed the pros and cons of various ML models (e.g.,

SVM, NB, and RFs). However, no clear conclusion has been drawn from this comparison. Can the authors provide a guideline of ML model selection for AD prediction using SNPs features? Various scenarios such as the number of SNP features, the number of samples, the presence of data issues such as missing values can be considered for the guideline. For example,
• Scenario A: 300 samples, 500 SNPs, which ML model should be used.
• Scenario B: 300 samples, 50,000 SNPs, which ML model should be used.
17. Although the neural network is not compared in this review, some NN-based AD risk prediction using SNPs data has been published recently. They can be added to the discussion section if they are not in the original date range for the paper search.
18. The curse of dimensionality is a major challenge in omics study (e.g., SNPs). Can the authors briefly discuss the challenges and opportunities from the ML perspective for AD prediction in the discussion section?
19. Although most studies use the ADNI dataset, it has some limitations, such as the small sample size (e.g., a few hundred) and population bias (e.g., most participants are Caucasian). As the quality of training data is essential for any ML models' success, can the authors provide insights on any alternative to the ADNI dataset? Such as alternative AD datasets or computational methods for data augmentation.
20. Based on the current discussion, can the authors summarize the challenges and opportunities of ML for AD risk prediction using genetic data? It would be helpful if the authors can provide some insights on the research priorities in this field (e.g., the collection of more data, the development of feature selection techniques, the improvements of classification techniques, or the development of model optimization techniques).

Minor suggestions:
1. Numbering of the subsection "3.5 Hyperparameter search" should be 3.6.
2. Numbering of the subsection "3.6 Descriptive statistics" should be 3.7.
3. Grammar: comma before "and" in a list of objects.
For example, "the type of models used, model development, and the validity of the reported results."

Reviewer 2: Dr. Javier De Velasco Oriol

Comments to the Author
The introduction mentions that the review will assess the ability of ML to predict AD using only genetic data, but in the next chapter the inclusion of multimodal data is included. This should be consistent. The abstract should also be modified to include the multimodal data. Furthermore, the Inclusion Criteria should also reflect this for clarity.

Further detail could be included into why an AUC of 0.8 using genetic data is an impressive metric due to the multifactorial complexity of the disease and the limits of the genetic component (Expanding into the value of multimodal analysis).

Including more insight into the missing heritability and the benefits of ML methods to solve multifactorial diseases should be considered.

In tables and comparisons (specially for AUC and ACC), the comments section could be improved by making it clearer which studies use multimodal data and which are SNP-only. Additionally, a lack of SNP-preprocessing or a large class-imbalance could also be included.

Editorial Office comments:

-Please supply a graphical abstract. A graphical abstract is a visual representation of the central finding or methodology of the paper. It will be displayed in the table of contents. Authors should strive to make the graphical abstract informative, interesting, visually appealing, and straightforward (no caption needed, no abbreviations except gene names). Please upload as a separate high resolution image file (tiff preferred). The technical requirements for the graphical abstracts are:
--size of the submitted image: 1200 pixels square at 300dpi
--font of the text: Arial 12-16 point (smaller fonts will not be legible online)

-Please include a paragraph about data availability in the 'Materials and Methods' section. We strongly encourage data sharing either as supplementary information or uploads to repositories which you can link in the paper.

Date Sent: 14-Apr-2021
